# Definition of a Novel Cuproptosis-Relevant lncRNA Signature for Uncovering Distinct Survival, Genomic Alterations, and Treatment Implications in Lung Adenocarcinoma

**DOI:** 10.1155/2022/2756611

**Published:** 2022-10-14

**Authors:** Zhuning Wang, Junqiao Yao, Tengyu Dong, Xing Niu

**Affiliations:** ^1^Shanghai Institute of Immunology, Department of Immunology and Microbiology, Shanghai Jiao Tong University School of Medicine, Shanghai 200025, China; ^2^Department of Surgical Oncology and General Surgery, The First Hospital of China Medical University, Key Laboratory of Precision Diagnosis and Treatment of Gastrointestinal Tumors (China Medical University), Ministry of Education, Shenyang, 110001 Liaoning, China; ^3^China Medical University, Shenyang, 110122 Liaoning, China

## Abstract

**Objective:**

Cuproptosis is a newly discovered copper-independent cell death modality, and limited evidence suggests the critical implications in human cancers. Nonetheless, the clinical impacts of cuproptosis-relevant lncRNAs in lung adenocarcinoma (LUAD) remain largely ill-defined. The present study was aimed at defining a cuproptosis-relevant lncRNA signature for LUAD and discuss the clinical utility.

**Methods:**

We collected transcriptome expression profiling, clinical information, somatic mutation, and copy number variations from TCGA-LUAD cohort retrospectively. The genetic alterations of cuproptosis genes were systematically assessed across LUAD, and cuproptosis-relevant lncRNAs were screened for defining a LASSO prognostic model. Genomic alterations, immunological and stemness features, and therapeutic sensitivity were studied with a series of computational approaches.

**Results:**

Cuproptosis genes displayed aberrant expression and widespread genomic alterations across LUAD, potentially modulated by m6A/m5C/m1A RNA modification mechanisms. We defined a cuproptosis-relevant lncRNA signature, with a reliable efficacy in predicting clinical outcomes. High-risk subset displayed higher somatic mutations, CNVs, TMB, SNV neoantigens, aneuploidy score, CTA score, homologous recombination defects, and intratumor heterogeneity, cytolytic activity, CD8+ T effector, and antigen processing machinery, proving that this subset might benefit from immunotherapy. Increased stemness indexes and activity of oncogenic pathways might contribute to undesirable prognostic outcomes for high-risk subset. Additionally, high-risk patients generally exhibited higher response to chemotherapeutic agents (cisplatin, etc.). We also predicted several small molecule compounds (GSK461364, KX2-391, etc.) for treating this subset.

**Conclusion:**

Accordingly, this cuproptosis-relevant lncRNA signature offers an efficient approach to identify and characterize diverse prognosis, genomic alterations, and treatment outcomes in LUAD, thus potentially assisting personalized therapy.

## 1. Introduction

Lung cancer is the leading cause of cancer-related deaths globally, with 1.8 million deaths in 2020 [1]. This disease leads to more deaths in contrast to breast, cervical, and colorectal cancer combined [2]. When asymptomatic early (I/II) disease with potentially curative treatment, >70% of cases diagnosed with advanced (III/IV) disease have rarely curative treatment, highly contributing to undesirable prognostic outcomes, with only 16.2% of cases diagnosed with the disease alive at 5 years [3]. About 85% of tumors are diagnosed as non-small-cell lung cancer (NSCLC), with lung adenocarcinoma (LUAD) as the most prevalent subtype [4]. The incidence of LUAD is increasing among current smokers, former smokers, and even nonsmokers [5]. LUAD tumors are highly heterogeneous, as proven by thousands of distinct mutations identified per cancer genome. Molecular genetic studies have revealed crucial driver oncogenes (TP53, TTN, KRAS, etc.) that may be druggable targets for newly developed therapeutic agents [6]. Driver mutation-based LUAD subtypes are of benefit in clinical settings and are becoming more frequent [7, 8]. Nonetheless, driver mutations themselves do not appear to be a direct determinant of malignancy, because subtypes on the basis of driver mutations alone cannot be sufficient to support prognostic outcomes. In addition, traditional histological subtypes are limited in determining histogenesis and malignancy.

Copper is an essential mineral nutrient, and its redox properties are both beneficial and toxic to cells [9]. It is an essential cofactor for enzymes mediating numerous key cellular functions, mitochondrial respiration, antioxidant defense, hormone, neurotransmitter, and pigment biosynthesis. [9]. However, dysregulated copper storage induces oxidative stress and cytotoxicity. Accumulated evidence demonstrates that copper is implicated in cellular proliferation and deaths and constitutes an exploitable dependency in LUAD [10]. For instance, copper is required for the autophagic kinases ULK1/2 to trigger LUAD [11]. Meta-analysis reveals that high serum copper level is linked to the increased risk of lung cancer [12]. Recently, Tsvetkov et al. discovered a novel form of cell death triggered by targeted accumulation of copper in mitochondria that induces lipoylated tricarboxylic acid cycle enzyme aggregation through directly binding to copper [13]. Thus, determining the molecular features of cuproptosis-relevant genes may assist in elucidating the heterogeneity of LUAD. Evidence suggests that cuproptosis-relevant genes exhibit aberrant expression and correlate to prognostic outcomes for clear cell renal cell carcinoma [14] and hepatocellular carcinoma [15]. The clinical impacts and mechanisms of cuproptosis in most cancer types remain largely ill-defined. Long noncoding RNAs (lncRNAs) are transcripts with >200 nucleotides but not translated into proteins, which modulate gene expression at multiple levels and participate in diverse biological processes, especially cell death mechanisms [16]. Thus, relationships of lncRNA with cuproptosis have potential implications in clinical research of LUAD. The present study defined a cuproptosis-relevant lncRNA signature that unveiled diverse prognostic outcomes, genomic alterations, and therapeutic implications in LUAD, which might assist guide personalized therapy.

## 2. Materials and Methods

### 2.1. Data and Resources

Transcriptome expression profiling (containing 535 LUAD and 59 normal tissues), somatic mutation data (mutation annotation format), and copy number variations (CNVs) were acquired from TCGA-LUAD cohort (https://portal.gdc.cancer.gov/). In addition, we collected the relevant clinical features (Supplementary table 1), with the removal of patients without survival information. The annotation file of Genome Reference Consortium Human Build 38 (GRCh38) from the GENCODE (version 36) was adopted to annotate the lncRNAs. The tumor mutation burden (TMB), single-nucleotide variation (SNV) neoantigens, aneuploidy score, cancer-testis antigen (CTA) score, homologous recombination defects, intratumor heterogeneity, immune checkpoints, and cytolytic activity- and IFN-*γ* response-relevant markers of TCGA-LUAD patients were acquired from the UCSC Xena database or previously published literature [17]. Stemness scores (mDNAsi and mRNAsi) were computed based on one-class logistic regression machine learning algorithm [18]. Supplementary figure 1 illustrates the schematic diagram of the study design.

### 2.2. Gene Set of Cuproptosis

Ten cuproptosis genes CDKN2A, FDX1, DLD, DLAT, LIAS, GLS, LIPT1, MTF1, PDHA1, and PDHB were acquired from a previously published literature [13]. Univariate-cox regression approach was adopted to analyze the relationships between cuproptosis genes and LUAD patients' overall survival (OS) utilizing survival package. At the transcriptional levels, associations between cuproptosis genes were estimated across LUAD utilizing Pearson's correlation test. The STRING website (https://string-db.org/) integrates all known and predicted interactions between proteins, covering physical and functional associations [19]. Interactions between proteins from cuproptosis genes were inferred through the STRING website by default, which were visualized through Cytoscape software [20].

### 2.3. Genetic Alteration Analysis

The mutation data were analyzed and visualized utilizing maftools package with default parameter settings [21]. CNVs were evaluated utilizing GISTIC2.0 for identifying arm-level alterations [22]. To quantify the overall fraction of genomic alterations, the fractions of genome altered, gained, and lost were computed, respectively.

### 2.4. Selection of Cuproptosis-Relevant lncRNAs

The relationships of cuproptosis genes with lncRNAs were computed through adopting Pearson's correlation test. Cuproptosis-relevant lncRNAs were screened in accordance with the criteria of |correlation efficient| > 0.4 and *p* value < 0.0001.

### 2.5. Definition of a Cuproptosis-Relevant lncRNA Signature

Prognostic cuproptosis-relevant lncRNAs with *p* < 0.05 were selected through univariate-cox regression approach, which were incorporated into least absolute shrinkage and selection operator (LASSO). This analysis was achieved utilizing glmnet package [23]. The optimal *λ* was selected to minimize the overfitting. The cuproptosis-relevant lncRNA signature was computed with the formula: risk score = ∑_*i*=1_^*n*^*Li*∗*βi*, where *n* indicates the number of prognostic cuproptosis-relevant lncRNAs; *Li* denotes the expression value of lncRNA *i*; and *βi* represents the regression coefficient of lncRNA *i*. With the median score as the cut-off value, we classified LUAD cases as low- and high-risk subsets. Principal component analysis (PCA) was adopted for verifying this classification.

### 2.6. Survival Analysis

Uni- and multivariate-cox regression methods were implemented for investigating the relationships of variables with OS. Hazard ratio, 95% confidence interval together with p-value were visualized through ggforest function. The OS, disease-free survival (DFS), disease-specific survival (DSS), and progression-free survival (PFS) between groups were estimated through Kaplan–Meier (K-M) curves. Significant differences were computed with log-rank test utilizing survival and survminer packages. Receiver operating characteristic curves (ROCs) were drawn to present the prediction efficacy with survivalROC package. LUAD patients were stratified by clinicopathological parameters, and survival analysis was conducted between low- and high-risk subsets in each subgroup.

### 2.7. Generation of a Nomogram Scoring System

A nomogram was created through incorporating the cuproptosis-relevant lncRNA signature and clinicopathological parameters utilizing rms package. In this scoring system, each variable was assigned a score, and the total score was computed through adding the scores from all variables in individuals. Calibration curves were drawn to evaluate the consistency of the nomogram-predicted and clinically observed OS outcomes.

### 2.8. Tumor-Infiltrating Immune Cells

TIMER [24], CIBERSORT [25], CIBERSORT-ABS, QUANTISEQ [26], MCP-counter [27], xCell [28], and EPIC [29] approaches were adopted to infer the abundance of tumor-infiltrating immune cells across LUAD.

### 2.9. Multiomics Analysis of Immunomodulators

Multiomics regulation landscape (including mRNA expression, CNV, and DNA methylation) of immunomodulators was analyzed. For surveying associations of mRNA expression of immunomodulators with DNA methylation, each methylation site was matched to the corresponding gene. Afterwards, we computed Spearman's correlations of each immunomodulator expression with the corresponding methylation sites. A single correlation value for each gene was acquired through averaging the correlation coefficients.

### 2.10. Gene Set Variation Analysis (GSVA)

GSVA was adopted to probe the potential biological function variations of low- and high-risk subsets [30]. The gene sets of Hallmark and known biological processes were acquired from the Molecular Signatures Database or previously published literature [31].

### 2.11. Prediction of Clinical Chemotherapeutic Response

Through adopting pRRophetic algorithm [32], clinical chemotherapeutic response was inferred on the basis of transcriptome expression profiling. This analysis was implemented through establishing statistical models from the gene expression and drug sensitivity information from the Genomics of Drug Sensitivity in Cancer (https://www.cancerrxgene.org) [33].

### 2.12. Prediction of Small Molecule Compounds

Expression profiles and somatic mutation data of the human cancer cell lines (CCLs) were downloaded from the Cancer Cell Line Encyclopedia project [34]. In addition, drug sensitivity data of the CCLs were collected from the CTRP and PRISM databases. Compounds with >20% missing data were removed before *K*-nearest neighbor imputation. Thereafter, pRRophetic package was applied to compute the AUC value of each compound [32].

### 2.13. Statistics

Appropriate R packages (version 4.0.2) were adopted for statistical analysis. A parametric test (Student's test or Pearson's correlation) was conducted for Gaussian data, while nonparametric test (Wilcoxon's test or Spearman's correlation) was used for non-Gaussian data. *p* < 0.05 indicated statistical significance.

## 3. Results

### 3.1. Landscape of Transcriptional, Genetic, and Epitranscriptomic Features and Prognostic Significance of Cuproptosis Genes across LUAD

**Figure 1:**
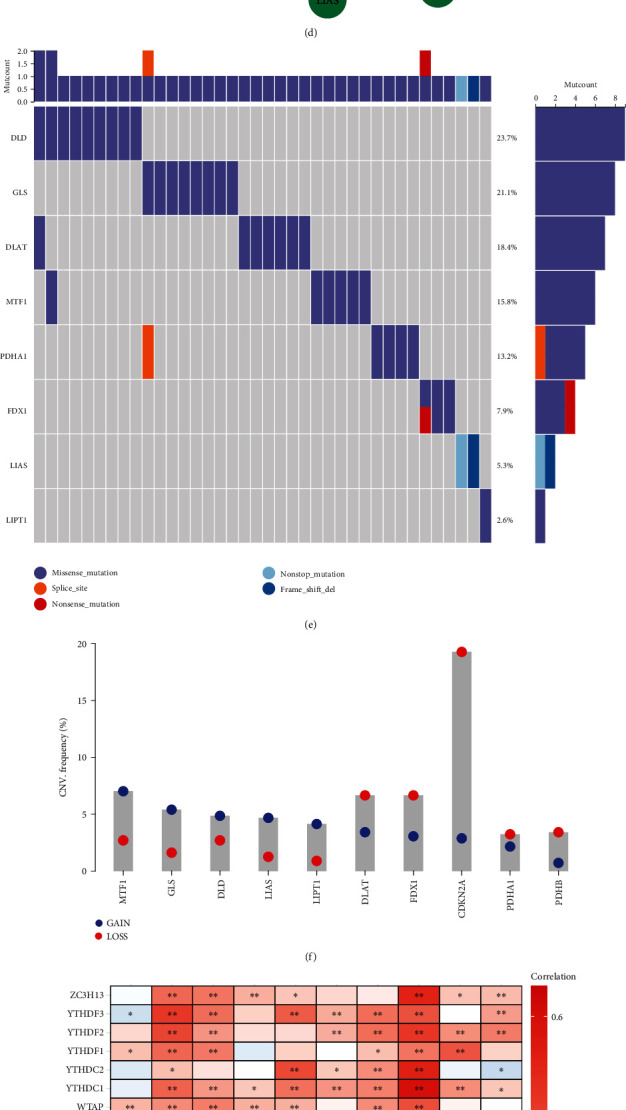
Landscape of transcriptional, genetic, and epitranscriptomic features and prognostic significance of cuproptosis genes across LUAD. (a) Heatmap visualizing the transcriptional levels of cuproptosis genes across normal and LUAD tissues. (b) Forest diagram for the univariate-cox regression results of cuproptosis genes with LUAD patients' OS. (c) Pearson's correlations between cuproptosis genes at the transcriptional levels. (d) Associations between proteins from cuproptosis genes through the STRING website. (e) Waterfall plot depicting the mutation events of cuproptosis genes across individual LUAD patients. Statistical plots of mutation events for each gene are displayed in the right panel. Variant classifications are marked by unique colors. (f) Bar chart for the CNV frequency of cuproptosis genes. Blue and red circles denote arm-level CNV gains and losses, respectively. (g–i) Heatmaps showing the interactions of m6A, m1A, and m5C modifiers with cuproptosis genes across LUAD. ^∗^*p* < 0.05; ^∗∗^*p* < 0.01.

The notable difference in transcriptional levels of cuproptosis genes was observed between normal and LUAD tissues, especially downregulated MTF1, LIAS, CDKN2A, PDHA1, and DLAT together with upregulated FDX1 in LUAD (Figure 1(a)). Through adopting univariate-cox regression approach, the relationships between cuproptosis genes and OS were assessed across LUAD. Among ten cuproptosis genes, DLD and PDHA1 served as risk factors of LUAD patients' OS (Figure 1(b)). Further, we observed the notable interactions between cuproptosis genes at the transcriptional levels, especially DLD and DLAT, LIAS and LIPT1, etc. (Figure 1(c)). In addition, associations between proteins from cuproptosis genes were found (Figure 1(d)). As illustrated in Figure 1(e), cuproptosis genes occurred the widespread mutations across LUAD, especially DLD (23.7%). Missense mutation was the most frequent variant classification of cuproptosis genes. MTF1, GLS, DLD, LIAS, and LIPT1 had higher frequencies of copy-number gains, with higher frequencies of copy-number losses for DLAT, FDX1, CDKN2A, PDHA1, and PDHB (Figure 1(f)). Epitranscriptomic features of cuproptosis genes were then evaluated. We observed that most m6A and m1A together with m5C modifiers were positively linked to cuproptosis genes at the transcriptional levels (Figures 1(g)–1(i)).

### 3.2. Identification of Cuproptosis-Relevant lncRNAs in LUAD

**Figure 2:**
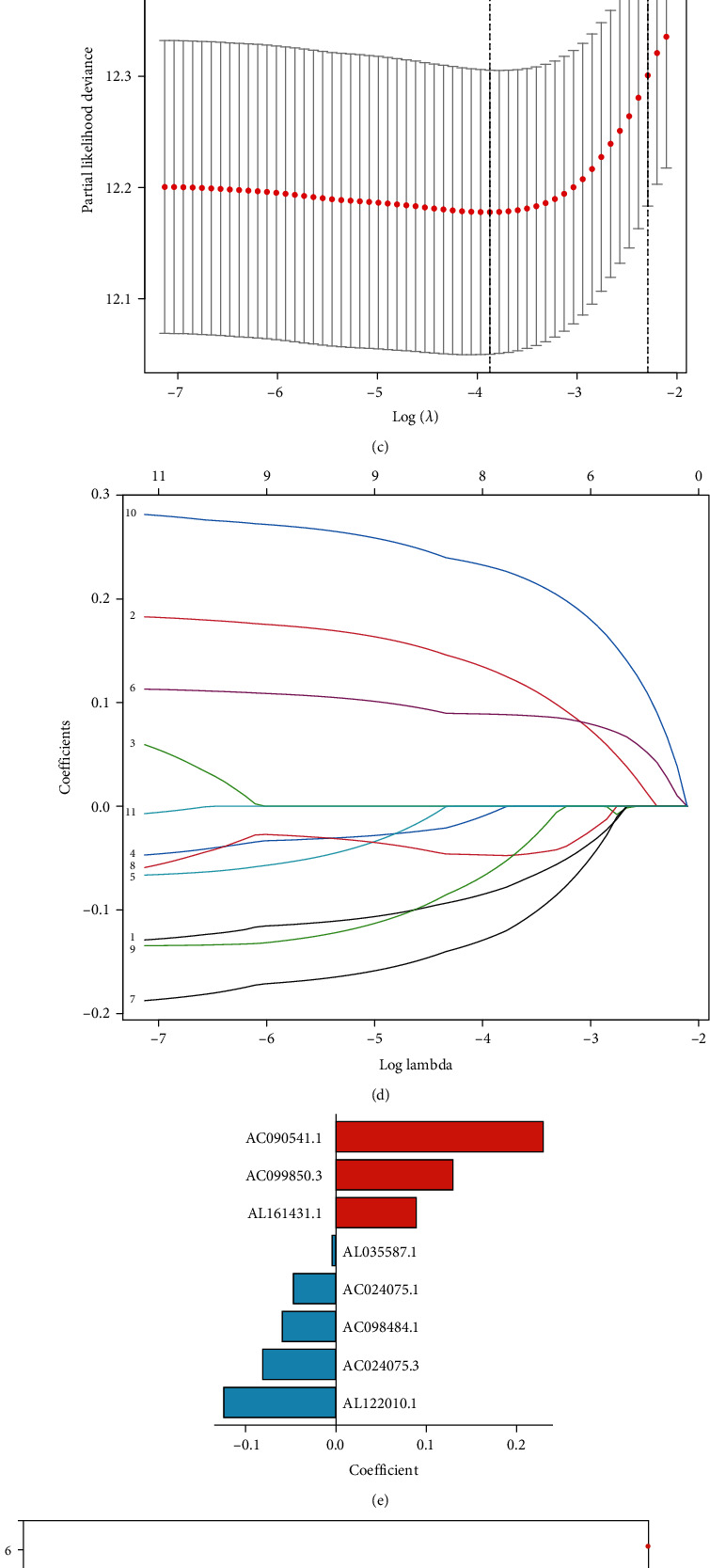
Definition of a cuproptosis-relevant lncRNA signature for LUAD. (a) Interactions of cuproptosis genes with lncRNAs across LUAD under the criteria of ∣correlation efficient | >0.4 and *p* value < 0.0001. (b) Forest diagram visualizing the prognostic cuproptosis-relevant lncRNAs. (c) Partial likelihood deviance under diverse log(*λ*) values for LASSO. (d) LASSO regression coefficients under diverse log(*λ*) values. (e) LASSO regression coefficients of each prognostic cuproptosis-relevant lncRNA. (f) Distribution of risk score derived from the cuproptosis-relevant lncRNA signature, survival time and status, and expression levels of prognostic cuproptosis-relevant lncRNAs. (g) PCA plots visualizing the difference between low- and high-risk subsets at the transcriptional levels.

On the basis of the criteria of |correlation efficient| > 0.4 and *p* value < 0.0001, fifty-five cuproptosis-relevant lncRNAs were determined in LUAD (Table 1, Figure 2(a)). Eleven cuproptosis-relevant lncRNAs were significantly linked to LUAD patients' OS outcomes, with AL122010.1, AL035587.1, AC098484.1, AC024075.3, AC008764.2, and AC024075.1 as protective factors and AC245041.2, AL161431.1, AC099850.3, AC090541.1, and LINC00592 as risk factors (Figure 2(b)).

### 3.3. Definition of a Cuproptosis-Relevant lncRNA Signature for LUAD

Prognostic cuproptosis-relevant lncRNAs were incorporated into LASSO to overcome the overfitting. On the basis of the optimal *λ* value, eight lncRNAs were applied for generating a prognostic signature (Figures 2(c) and 2(d)).  Figure 2(e) depicts the LASSO coefficient of each lncRNA. The risk score was computed in line with the formula: risk score = AL122010.1 level^∗^ (−0.124158664) + AC024075.3 level^∗^ (−0.081144659) + AC098484.1 level^∗^ (−0.059433042) + AC024075.1 level^∗^ (−0.047301757) + AL035587.1 level^∗^ (−0.004423536) + AL161431.1 level^∗^ 0.088703142 + AC099850.3 level^∗^ 0.129487682 + AC090541.1 level^∗^ 0.22936017. Thereafter, we classified LUAD patients as low- and high-risk subsets (Figure 2(f)). More dead cases were observed in high-risk subset. PCA confirmed the prominent difference between two subsets (Figure 2(g)).

### 3.4. The Excellent Efficacy of the Cuproptosis-Relevant lncRNA Signature in Prognosis Prediction

**Figure 3:**
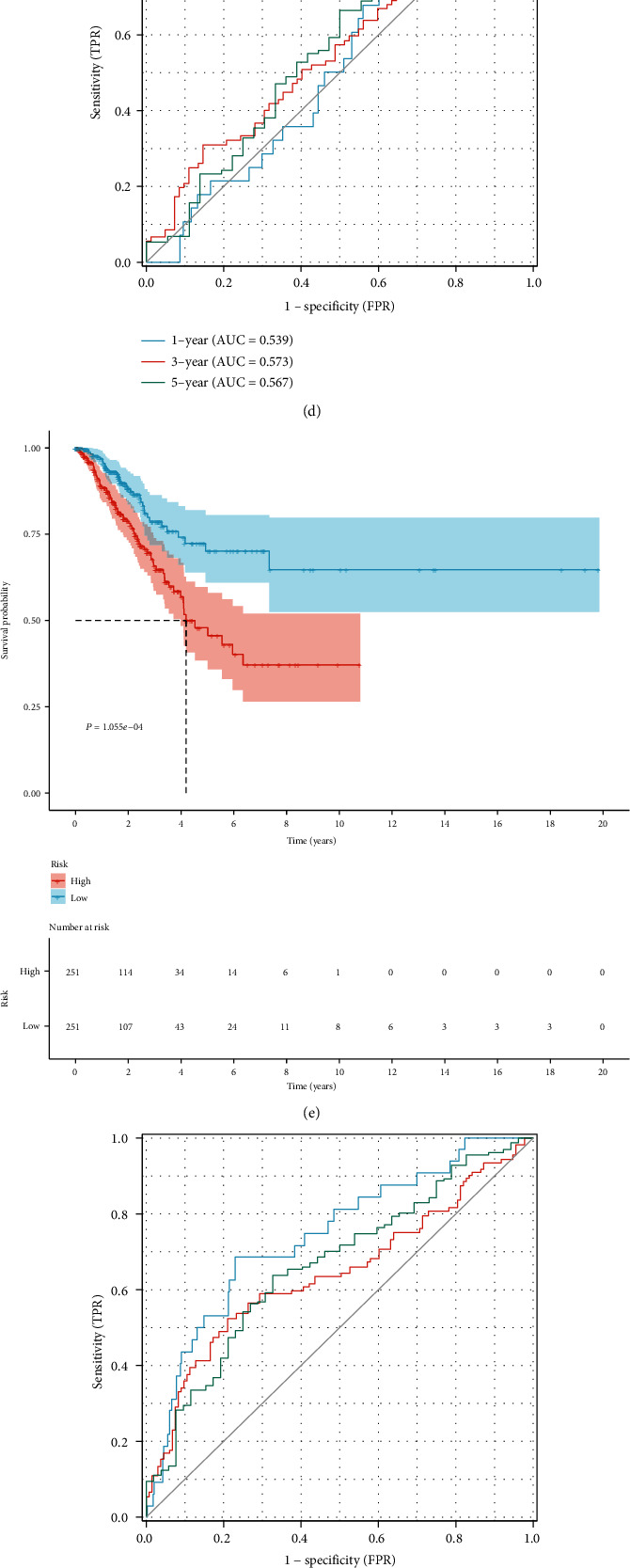
The excellent efficacy of the cuproptosis-relevant lncRNA signature in predicting LUAD prognosis. (a, b) K-M curves of OS between low- and high-risk subsets and ROC curves at 1- and 3-year OS, together with 5-year OS. (C, D) K-M curves of DFS between subsets, and ROC curves at 1- and 3-year DFS, together with 5-year DFS. (e, f) K-M curves of DSS between subsets and ROC curves at 1- and 3-year DSS, together with 5-year DSS. (h, h) K-M curves of PFS between subsets and ROC curves at 1- and 3-year PFS, together with 5-year PFS. (i) K-M curves of OS between two subsets stratified by the median values of expression of prognostic cuproptosis-relevant lncRNAs.

In contrast to low-risk subset, poorer OS outcomes were observed in high-risk subset (Figure 3(a)). ROC curves were plotted to estimate the efficacy of the cuproptosis-relevant lncRNA signature in OS prediction. The AUC values at 1-, 3-, and 5-year OS were all >0.6, indicating the high sensitivity and specificity of this signature in predicting OS outcomes (Figure 3(b)). In addition, the risk score reliably enabled to differentiate DFS and DSS together with PFS outcomes of low- and high-risk subsets (Figures 3(c)–3(h)). The above evidence suggested that the cuproptosis-relevant lncRNA signature might possess the potential in reflecting LUAD patients' clinical outcomes and benefits. Further, the prognostic value of each prognostic cuproptosis-relevant lncRNA was assessed. As illustrated in Figure 3(i), upregulated AC024075.1, AC024075.3, AC098484.1, AL035587.1, and AL122010.1 were linked to more favorable OS outcomes, with poorer OS for upregulated AC090541.1, AC099850.3, and AL161431.1.

### 3.5. The Sensitivity and Independency of the Cuproptosis-Relevant lncRNA Signature in Predicting LUAD Prognosis

**Figure 4:**
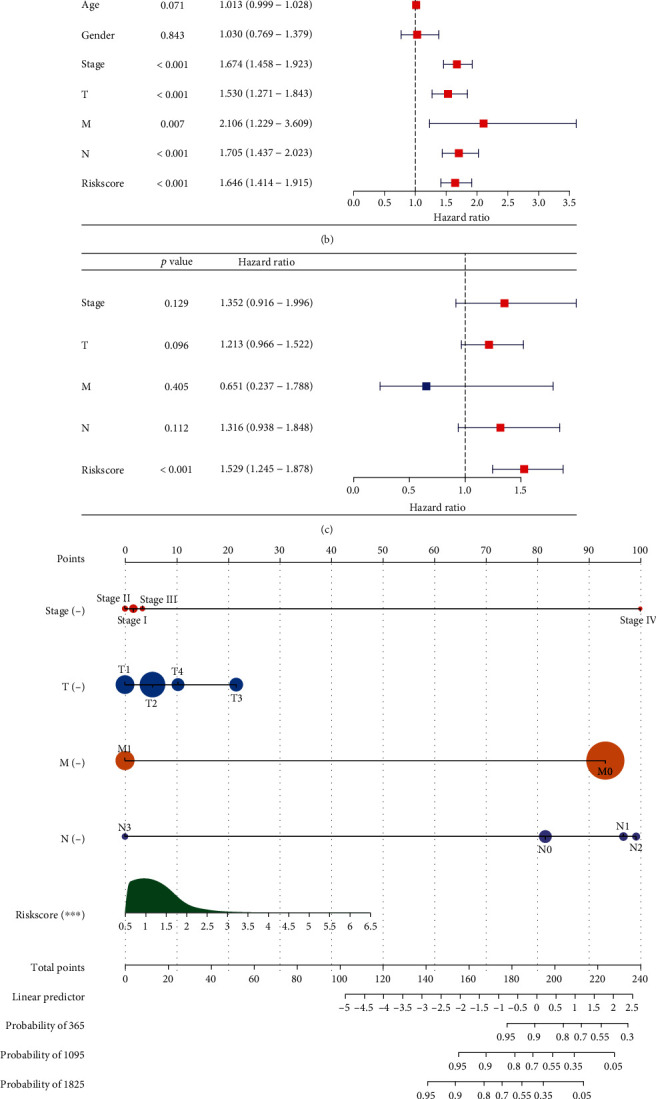
Evaluation of the sensitivity and independency of the cuproptosis-relevant lncRNA signature in prognosis prediction and generation of a nomogram for LUAD. (a) K-M curves of OS between low- and high-risk subsets in diverse subgroups stratified by known clinicopathological parameters. (b, c) Forest diagrams depicting uni- and multivariate-cox regression results of the relationships of the cuproptosis-relevant lncRNA signature and clinicopathological parameters with OS outcomes. (d) Generation of a nomogram scoring system incorporating the cuproptosis-relevant lncRNA signature together with clinicopathological parameters (histological stage, TNM). Each variable corresponds to a point, and total point refers to the total score obtained by adding up the corresponding points of all variables. (e) Calibration curves illustrating the nomogram scoring system-estimated and clinically observed 1-, 3-, and 5-year OS outcomes.

To further appraise the prediction efficacy of the cuproptosis-relevant lncRNA signature, LUAD patients were stratified by distinct clinicopathological parameters (age, sex, histological stage, and TNM). In each subgroup, high-risk subset exhibited more unfavorable OS outcomes (Figure 4(a)). Uni- and multivariate cox regression results confirmed that the cuproptosis-relevant lncRNA signature was an independent risk factor of LUAD (Figures 4(b) and 4(c)).

### 3.6. Generation of a Cuproptosis-Relevant lncRNA Signature-Based Nomogram Scoring System

To facilitate clinical application of the cuproptosis-relevant lncRNA signature, we created a nomogram that incorporated this signature together with clinicopathological parameters (histological stage, TNM) for individuals, as illustrated in Figure 4(d). Calibration curves proved the high consistency between this nomogram scoring system-estimated and clinically observed 1-, 3-, and 5-year OS (Figure 4(e)).

### 3.7. Genomic Alteration Features of the Cuproptosis-Relevant lncRNA Signature

**Figure 5:**
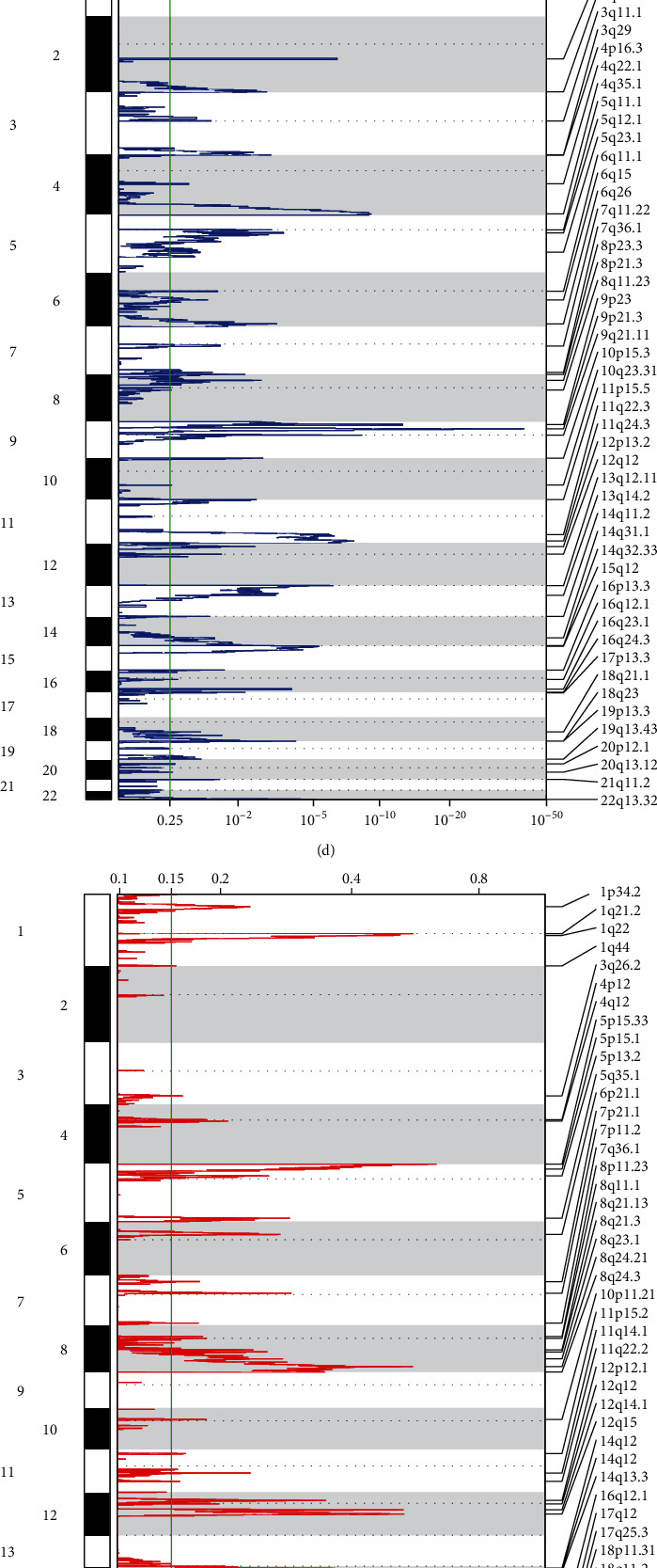
Genomic alteration features of the cuproptosis-relevant lncRNA signature. (a) Waterfall plot illustrating the first 20 mutated genes in high-risk subset. Statistical plots of mutation events for each mutated gene are exhibited in the right panel. Variant classifications are labeled by unique colors. (b) Waterfall plot depicting the first 20 mutated genes in low-risk subset. (c, d) Significant focal copy-number gains (red) and losses (blue) in high-risk subset. (e, f) Significant focal copy-number gains and losses in low-risk subset. (g) Distribution of fraction of genome altered, lost, and gained in low- and high-risk subsets. (h–m) Comparison of TMB, SNV neoantigens, aneuploidy score, CTA score, homologous recombination defects, and intratumor heterogeneity between two subsets. ^∗^*p* < 0.05; ^∗∗∗^*p* < 0.001; ^∗∗∗∗^*p* < 0.0001.

Waterfall plots displayed that notably higher somatic mutation frequencies (TP53, TTN, CSMD3, etc.) were observed in high- than low-risk subset (Figures 5(a) and 5(b)). In addition, GISTIC2.0 was adopted for delineating the significant CNVs of each subset. Consequently, high-risk subset had more copy-number gains and losses in contrast to low-risk subset (Figures 5(c)–5(f)). As expected, there were higher fractions of genome altered, lost, and gained in high-risk subset (Figure 5(g)). The above findings unveiled the diverse genomic alteration preferences in two subsets.

### 3.8. Implication of the Cuproptosis-Relevant lncRNA Signature in Immunotherapy

**Figure 6:**
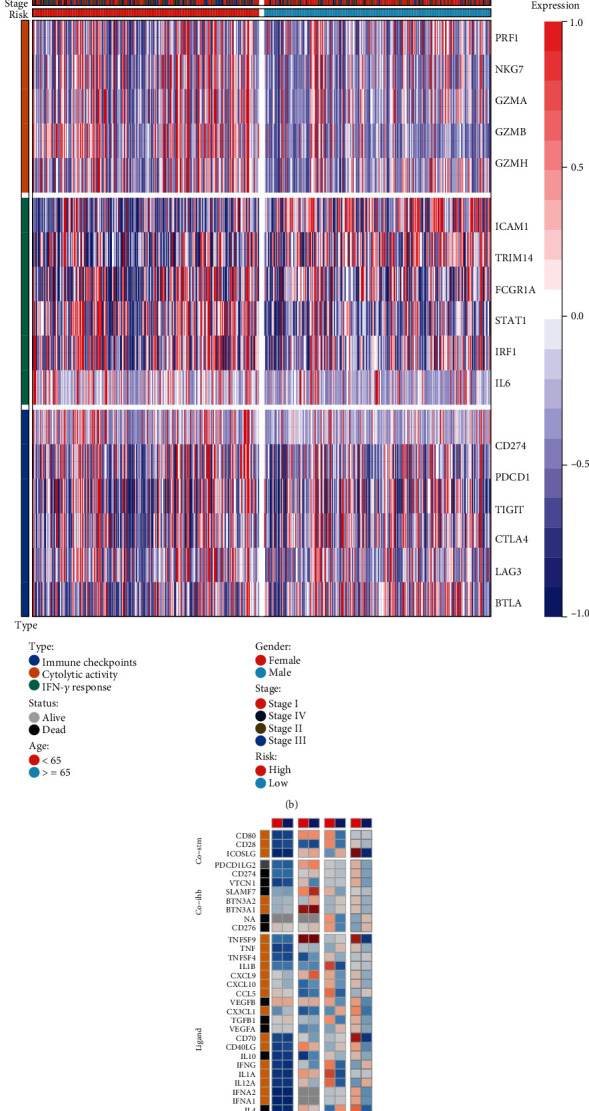
Implication of the cuproptosis-relevant lncRNA signature in immunotherapy. (a) The abundance levels of tumor-infiltrating immune cells calculated by diverse computational approaches in low- and high-risk subsets. (b) Expression patterns of immune checkpoints, cytolytic activity- and IFN-*γ* response-relevant markers across low- and high-risk subsets. (c) Landscape of mRNA expression, expression vs. methylation (mRNA expression correlated to DNA-methylation *β* values), amplification, and deletion frequencies for immunomodulators in low- and high-risk subsets.

Immunogenomic indicators were measured across LUAD. Higher TMB, SNV neoantigens, aneuploidy score, CTA score, homologous recombination defects, and intratumor heterogeneity were observed in high-risk subset (Figures 5(h)–5(m)). Seven computational approaches were adopted for inferring the abundance levels of tumor-infiltrating immune cells across LUAD. The results derived from most approaches showed the widespread heterogeneity in immune cells within the tumor microenvironment between low- and high-risk subsets (Figure 6(a)). High-risk subset exhibited higher expression of cytolytic activity-relevant markers (Figure 6(b)). However, modest differences in immune checkpoints and IFN-*γ* response-relevant markers were observed between subsets. In Figure 6(c), immunomodulators had little differences in two subsets. On the basis of above evidence, high-risk LUAD might benefit from immunotherapy.

### 3.9. Biological State, Process, and Stemness Features of the Cuproptosis-Relevant lncRNA Signature

**Figure 7:**
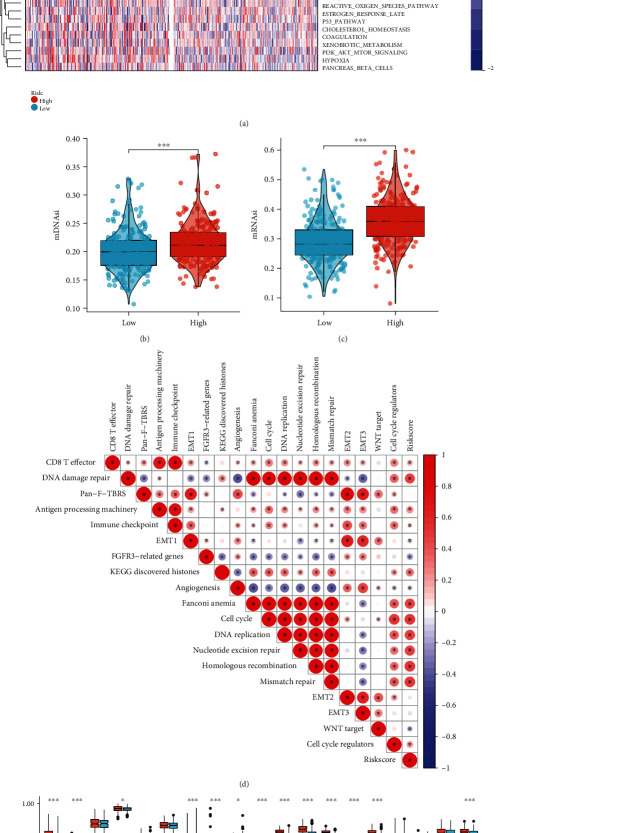
Biological state, process, and stemness features of the cuproptosis-relevant lncRNA signature. (a) Distribution of the variations of Hallmark gene set activity across low- and high-risk subsets. (b, c) Comparison of mDNAsi and mRNAsi between two subsets. (d) Correlations of risk score with the activity of known biological processes. (e) Comparison of the activity of known biological processes between two subsets. ^∗^*p* < 0.05; ^∗∗∗^*p* < 0.001.

Among the 50 Hallmark gene sets, oncogenic pathways (mTORC1, MYC, E2F, glycolysis, etc.) exhibited higher activity in high- than low-risk subset (Figure 7(a)). In addition, increased stemness indexes mDNAsi and mRNAsi were observed in high-risk subset (Figures 7(b) and 7(c)). Further, the risk score displayed notably positive interactions with CD8+ T effector, antigen processing machinery, nucleotide metabolism, and cell cycle (Figures 7(d) and 7(e)). The above data revealed the reasons for the poor prognosis of high-risk population.

### 3.10. Clinical Therapeutic Benefit of the Cuproptosis-Relevant lncRNA Signature for Chemotherapeutic Agents and Small Molecule Compounds

**Figure 8:**
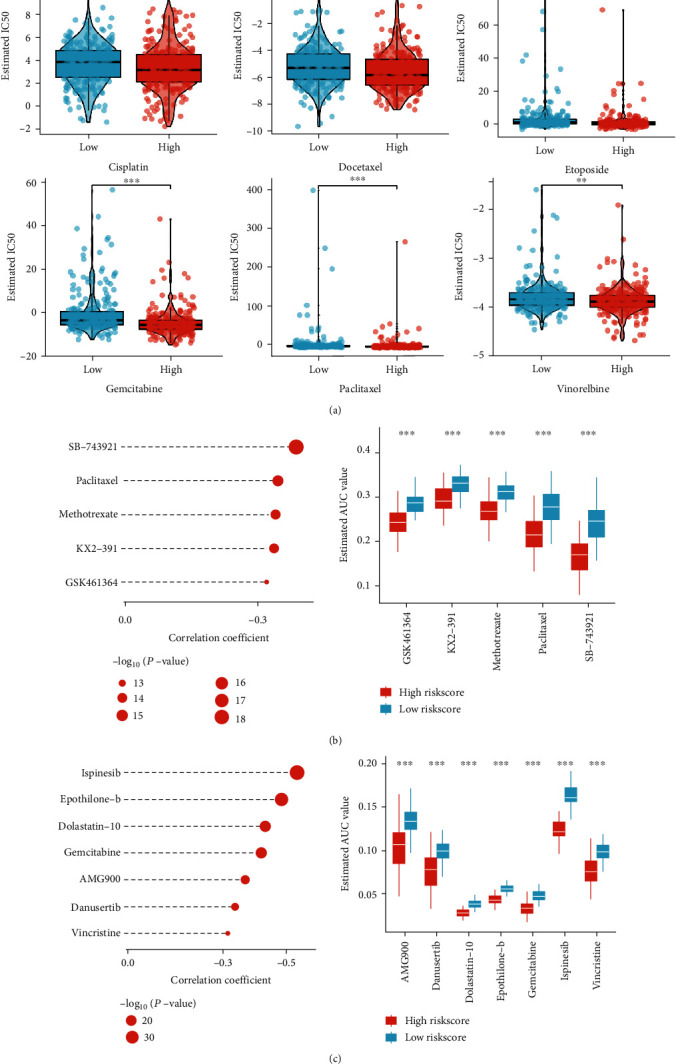
Clinical therapeutic benefit of the cuproptosis-relevant lncRNA signature for chemotherapy and small molecule compounds. (a) Boxplots displaying the estimated clinical chemotherapeutic sensitivity in low- and high-risk subsets. (b) Correlations of risk score with AUC values of CTRP-derived small molecule compounds (left) and comparison of AUC values between low- and high-risk subsets (right). (c) Associations of risk score with AUC values of PRISM-derived small molecule compounds (left) and comparison of AUC values between low- and high-risk subsets (right). ^∗∗^*p* < 0.01; ^∗∗∗^*p* < 0.001.

Lower IC50 values of chemotherapeutic agents (cisplatin, docetaxel, etoposide, gemcitabine, paclitaxel, and vinorelbine) were observed in high-risk subset than in low-risk subset (Figure 8(a)). On the basis of this evidence, high-risk patients generally displayed higher response to chemotherapy. Considering poor prognostic outcomes of high-risk patients, the current study predicted small molecule compounds for aiming at this patient subset. Consequently, five CTRP-derived small molecule compounds (GSK461364, KX2-391, methotrexate, paclitaxel, and SB-743921; Figure 8(b)) together with PRISM-derived small molecule compounds (AMG900, danusertib, dolastatin-10, epothilone-b, gemcitabine, ispinesib, and vincristine; Figure 8(c)) were inferring for high-risk patients.

## 4. Discussion

Prominent treatment improvement, immunotherapy, etc. has resulted in improved prognosis for a small number of patients with advanced (stage IV) LUAD [2]. Nonetheless, clinical outcomes of most patients remain undesirable. Cuproptosis is a newly discovered copper-dependent regulated cell death that depends upon mitochondrial respiration, unlike known death mechanisms (ferroptosis, necroptosis, pyroptosis, etc.) [13]. Cuproptosis genes displayed aberrant expression and widespread genomic alterations across LUAD, which were potentially modulated by m6A/m5C/m1A RNA modification mechanisms, proving the potential impacts of cuproptosis in LUAD.

Previously, cuproptosis-related lncRNA signatures have been conducted for hepatocellular carcinoma [15], colon adenocarcinoma [35], and osteosarcoma [36]. Herein, we defined a cuproptosis-relevant lncRNA signature (comprising AC024075.1, AC024075.3, AC098484.1, AL035587.1, AL122010.1, AC090541.1, AC099850.3, and AL161431.1) for LUAD through adopting LASSO computational approach, which might be a potential prediction tool of OS that was independent of other clinicopathological parameters. An individualized nomogram based on the cuproptosis-relevant lncRNA signature together with clinicopathological parameters (histological stage, TNM) was developed for predicting OS outcomes, which might act as a supplementary tool to assess the probabilities of OS in LUAD. Surgery provides the optimal prognostic outcomes for patients with primary NSCLC, though long-term survival following surgery is still low. Recurrence of postoperative NSCLC occurs within the first 5 years, ranging 20%~75% of cases. Most recurrences (>80%) occur in the first 2 years following surgery [37]. The cuproptosis-relevant lncRNA signature potentially predicted LUAD recurrence, with poorer DFS for high-risk subset.

Among the lncRNAs in the cuproptosis-relevant lncRNA signature, upregulated AC024075.1, AC024075.3, AC098484.1, AL035587.1, and AL122010.1 were associated with better OS outcomes, with worse OS for upregulated AC090541.1, AC099850.3, and AL161431.1. Limited evidence has proven the roles of above lncRNAs in human cancers. For instance, AC098484.1 is found to correlate to autophagy of clear cell renal cell carcinoma [38]. AL035587.1 is potentially modulated by m6A, m5C, and m1A RNA modifications in head and neck squamous cell carcinoma [39]. AL122010.1 is an immune- [40], stemness- [41], autophagy- [42], and m6A-relevant lncRNA [43] that enables to predict survival outcomes breast cancer. AC099850.3 is upregulated in LUAD and correlated to advanced stage, undesirable prognostic outcomes together with immune infiltration [44]. Evidence proves that AC099850.3 is a necroptosis- [45] and ferroptosis-related lncRNA [46] in cancer patients. Necroptosis and ferroptosis are newly discovered programmed cell deaths recently [47]. AL161431.1 acts as an autophagy- and prognosis-relevant lncRNA in NSCLC [48], with epigenetically aberrant regulation in LUAD [49] and pancreatic adenocarcinoma [50]. In addition, AL161431.1 is linked to hypoxia status and immune microenvironment of LUAD [51]. Suppression of AL161431.1 results in enhanced cell death and cell cycle arrest in pancreatic cancer [52].

Tumor heterogeneity can be attributed to genetic and nongenetic alterations. Genomic instability (somatic mutations, CNVs, etc.) is regarded as hallmark of cancer, resulting in genetic aberrations. Genome and transcriptome unveiled that LUAD tumors exhibit increased TMB in contrast to other cancer types [53]. In addition, evidence supports that LUAD tumors arise from progenitor clones with early genomic alterations that trigger tumorigenesis. Our evidence proved that high-risk subset occurred higher frequencies of somatic mutations and CNVs, together with increased TMB. Immunotherapy may assist the immune system recognize and attack tumor cells. The major targets of ICB therapy are PD-L1 and PD1 together with CTLA4. In contrast to conventional treatment, ICB is notably limited to only one-third of patients responding to ICB. Theoretically, high TMB enhances the probability of tumor neoantigen generation, thus improving immune recognition and tumor cell killing [54]. In addition, genomic and transcriptional changes in first-line chemotherapy have potential adverse effects on subsequent immunotherapy in NSCLC [55]. Higher SNV neoantigens, aneuploidy score, CTA score, homologous recombination defects, and intratumor heterogeneity were observed in high-risk subset. Cytolytic activity, CD8+ T effector, and antigen processing machinery exhibited higher activity in this subset. Altogether, our evidence proved that high-risk patients were more sensitive to immunotherapy.

Most LUAD patients die of cancer metastasis and treatment resistance [56]. Cancer stem cells possess the abilities of normal stem cells, especially drug resistance, metastasis, immune escape, etc. [57]. Active bronchioalveolar stem cells are a potential origin of LUAD cells [58]. We computed the stemness indices of LUAD, and observed higher mDNAsi and mRNAsi in high-risk subset. Cisplatin-based adjuvant chemotherapy is still the standard of care for patients with resected stage II or III NSCLC [59]. High-risk patients generally displayed higher response to chemotherapeutic agents (cisplatin, docetaxel, etoposide, gemcitabine, paclitaxel, and vinorelbine). One goal of precision therapy is to determine subpopulations of LUAD patients who may benefit from a specific treatment strategy. Hence, to find druggable targets together with compounds to mitigate oncogenic signaling remains a key challenge in LUAD. Through combining drug development and pharmacogenomics with molecular characterization of LUAD, we screened five CTRP-derived small molecule compounds (GSK461364, KX2-391, methotrexate, paclitaxel, and SB-743921) and PRISM-derived small molecule compounds (AMG900, danusertib, dolastatin-10, epothilone-b, gemcitabine, ispinesib, and vincristine) for potentially treating high-risk patients.

A few disadvantages in this study are pointed out. Firstly, the LUAD lncRNA datasets are limited. The prediction performance of the cuproptosis-relevant lncRNA signature in LUAD prognosis will be further externally verified in larger cohorts. Secondly, due to the lack of LUAD patients with immunotherapy currently, we investigated only the relationships of the cuproptosis-relevant lncRNA signature with immune response indicators, which is required for further verification, with in-depth investigations for practical application of risk score into clinical cohorts. Thirdly, more experimental verifications are required for comprehensively interpreting risk score as well as the therapeutic effects of small molecule compounds.

## 5. Conclusion

In summary, the current study defined the cuproptosis-relevant lncRNA signature that unveiled diverse prognostic outcomes, genomic alterations, and treatment outcomes across LUAD, which might potentially guide clinical management and personalized treatment.

## Figures and Tables

**Table 1:** Fifty-five cuproptosis-relevant lncRNAs in LUAD.

Cuproptosis gene	LncRNA	Correlation	*p* value
MTF1	AC024075.3	0.4368	2.07*E* − 25
FDX1	SLFNL1-AS1	0.5978	3.31*E* − 51
MTF1	LINC02035	0.4189	2.69*E* − 23
LIAS	AC008966.1	0.4154	6.71*E* − 23
DLD	AC099850.3	0.4306	1.15*E* − 24
DLAT	AC099850.3	0.4479	8.90*E* − 27
FDX1	AC005532.1	0.6145	8.51*E* − 55
DLAT	AC115837.1	0.4322	7.45*E* − 25
FDX1	AC110619.1	0.4223	1.07*E* − 23
GLS	AC019080.5	0.4164	5.11*E* − 23
FDX1	AC092809.2	0.8266	3.64*E* − 130
MTF1	NORAD	0.443	3.62*E* − 26
MTF1	AC008764.2	0.4138	1.00*E* − 22
MTF1	AL049840.3	0.4292	1.71*E* − 24
GLS	AC008063.1	0.5563	3.50*E* − 43
MTF1	AC073046.1	0.5224	2.09*E* − 37
MTF1	AL035587.1	0.4301	1.31*E* − 24
FDX1	AC245041.2	0.6145	8.57*E* − 55
GLS	AC012020.1	0.4377	1.62*E* − 25
LIPT1	OSER1-DT	0.4231	8.87*E* − 24
DLAT	AC005288.1	0.4073	5.34*E* − 22
MTF1	AC005288.1	0.428	2.34*E* − 24
GLS	AL354989.1	0.4637	8.08*E* − 29
MTF1	AC073073.2	0.4027	1.69*E* − 21
GLS	SP2-AS1	0.4495	5.54*E* − 27
MTF1	AC005034.5	0.4184	3.01*E* − 23
MTF1	LINC01128	0.4058	7.79*E* − 22
MTF1	AL049840.2	0.4522	2.57*E* − 27
MTF1	AC012313.5	0.4349	3.50*E* − 25
FDX1	AL161431.1	0.607	3.61*E* − 53
LIAS	LINC00467	0.4128	1.31*E* − 22
GLS	AL158166.2	0.4357	2.86*E* − 25
MTF1	AL122010.1	0.4016	2.24*E* − 21
MTF1	AC024075.1	0.4938	5.20*E* − 33
MTF1	AL731577.2	0.523	1.67*E* − 37
FDX1	AC026785.3	0.4953	3.11*E* − 33
GLS	PRR7-AS1	0.4248	5.65*E* − 24
MTF1	AC108010.1	0.4496	5.36*E* − 27
FDX1	LINC00189	0.5468	1.72*E* − 41
LIAS	SRP14-AS1	0.4359	2.70*E* − 25
MTF1	AC098484.1	0.45	4.86*E* − 27
FDX1	AC090541.1	0.6222	1.67*E* − 56
MTF1	LINC01963	0.4266	3.41*E* − 24
GLS	AC000123.1	0.4008	2.69*E* − 21
CDKN2A	MELTF-AS1	0.4042	1.17*E* − 21
GLS	PPP1R12A-AS1	0.4023	1.86*E* − 21
MTF1	AC068768.1	0.4746	2.77*E* − 30
MTF1	Z68871.1	0.4857	7.66*E* − 32
MTF1	AC105206.2	0.4015	2.28*E* − 21
FDX1	LINC00592	0.4133	1.15*E* − 22
MTF1	AP003486.1	0.4943	4.43*E* − 33
MTF1	STARD7-AS1	0.4353	3.16*E* − 25
GLS	AC005540.1	0.5446	4.17*E* − 41
MTF1	AL160006.1	0.5039	1.65*E* − 34
GLS	AL158166.1	0.4251	5.13*E* − 24

## Data Availability

The datasets analyzed during the current study are available from the corresponding author on reasonable request.

## Abbreviations

NSCLC: Non-small-cell lung cancer
LUAD: Lung adenocarcinoma
CNVs: Copy number variations
TMB: Tumor mutation burden
SNV: Single nucleotide variation
CTA: Cancer-testis antigen
OS: Overall survival
LASSO: Least absolute shrinkage and selection operator
PCA: Principal component analysis
DFS: Disease-free survival
DSS: Disease-specific survival
PFS: Progression-free survival
K-M: Kaplan–Meier
ROCs: Receiver operating characteristic curves
GSVA: Gene set variation analysis
CCLs: Cancer cell lines.

## Supplementary Materials

Supplementary 1 Supplementary figure 1: the schematic diagram of the study design.

Supplementary 2 Supplementary table 1: the clinical information of TCGA-LUAD cohort.
