# Supplementary figures and images for: Definition of a Novel Cuproptosis-Relevant lncRNA Signature for Uncovering Distinct Survival, Genomic Alterations, and Treatment Implications in Lung Adenocarcinoma
Source: J Immunol Res. 2022 Oct 14;2022:2756611. doi: 10.1155/2022/2756611 (PMC9587678; doi:10.1155/2022/2756611)

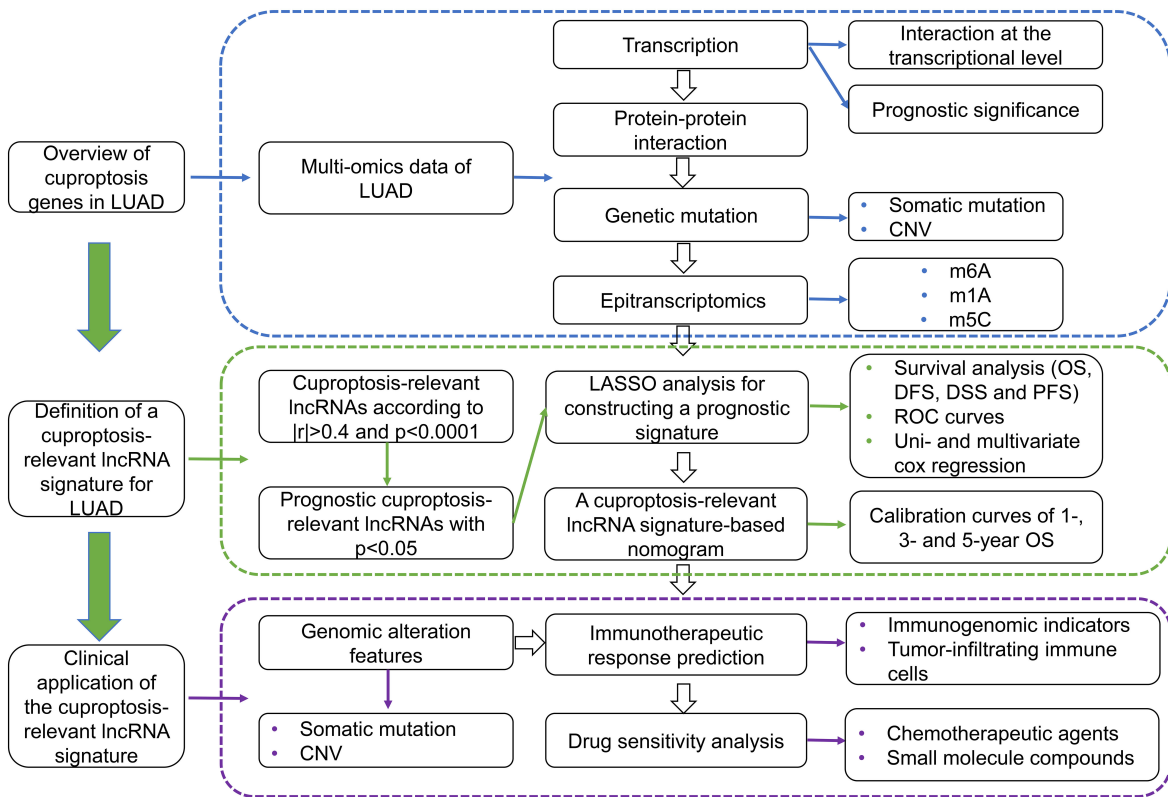

Supplement: Supplementary 1 — Supplementary figure 1: the schematic diagram of the study design. [file 2756611.f1.pdf]
