# Supplementary material for: Definition of a Novel Cuproptosis-Relevant lncRNA Signature for Uncovering Distinct Survival, Genomic Alterations, and Treatment Implications in Lung Adenocarcinoma
Source: J Immunol Res. 2022 Oct 14;2022:2756611. doi: 10.1155/2022/2756611 (PMC9587678; doi:10.1155/2022/2756611)
Supplement: Supplementary 2 — Supplementary table 1: the clinical information of TCGA-LUAD cohort. [file 2756611.f2.pdf]

Supplementary table 1. The clinical information of TCGA-LUAD cohort.

ID	Age	Gender	Stage	T	M	N
TCGA-35-4123-01	<65	Male	Stage I-II	T1-2	M0	N0
TCGA-49-AARO-01	<65	Female	Stage I-II	T1-2	NA	N0
TCGA-86-8076-01	<65	Male	Stage I-II	T1-2	M0	N0
TCGA-97-A4M6-01	<65	Female	Stage I-II	T1-2	M0	N0
TCGA-L4-A4E5-01	<65	Female	Stage I-II	T1-2	M0	N0
TCGA-93-A4JQ-01	<65	Male	Stage I-II	T1-2	NA	N0
TCGA-NJ-A4YF-01	<65	Female	Stage I-II	T1-2	M0	N0
TCGA-99-8028-01	<65	Female	Stage I-II	T1-2	M0	N0
TCGA-49-AARE-01	<65	Female	Stage I-II	T1-2	NA	N0
TCGA-97-A4M1-01	<65	Female	Stage I-II	T1-2	M0	N0
TCGA-4B-A93V-01	<65	Female	Stage I-II	T1-2	M0	N0
TCGA-44-7672-01	<65	Female	Stage I-II	T1-2	M0	N0
TCGA-80-5608-01	<65	Female	Stage I-II	T1-2	M0	N0
TCGA-55-8507-01	<65	Male	Stage I-II	T1-2	NA	N0
TCGA-97-8552-01	<65	Female	Stage I-II	T1-2	NA	N0
TCGA-55-6980-01	<65	Male	Stage I-II	T1-2	M0	N0
TCGA-55-8089-01	<65	Male	Stage I-II	T1-2	M0	N0
TCGA-55-8206-01	<65	Male	Stage I-II	T1-2	M0	N0
TCGA-49-AARN-01	<65	Female	Stage I-II	T1-2	NA	N0
TCGA-67-6216-01	<65	Female	Stage I-II	T1-2	M0	N0
TCGA-MN-A4N4-01	<65	Male	Stage I-II	T1-2	M0	N0
TCGA-49-AAR0-01	<65	Male	Stage I-II	T1-2	NA	N0
TCGA-64-1676-01	<65	Male	Stage I-II	T1-2	M0	N0
TCGA-73-7498-01	<65	Female	Stage I-II	T1-2	M0	N0
TCGA-97-7553-01	<65	Female	Stage I-II	T1-2	NA	N0
TCGA-83-5908-01	<65	Female	Stage I-II	T1-2	M0	N0
TCGA-91-6840-01	<65	Female	Stage I-II	T1-2	M0	N0
TCGA-44-6778-01	<65	Male	Stage I-II	T1-2	NA	N0
TCGA-50-7109-01	<65	Male	Stage I-II	T1-2	M0	N0
TCGA-44-3918-01	<65	Female	Stage I-II	T1-2	M0	N0
TCGA-44-6148-01	<65	Male	Stage I-II	T1-2	M0	N0
TCGA-44-6776-01	<65	Female	Stage I-II	T1-2	NA	N0
TCGA-L9-A444-01	<65	Female	Stage I-II	T1-2	NA	N0
TCGA-55-8097-01	<65	Female	Stage I-II	T1-2	NA	N0
TCGA-55-6543-01	<65	Female	Stage I-II	T1-2	NA	N0
TCGA-55-7570-01	<65	Male	Stage I-II	T1-2	NA	N0
TCGA-64-1681-01	<65	Female	Stage I-II	T1-2	M0	N0
TCGA-99-8032-01	<65	Male	Stage I-II	T1-2	M0	N0
TCGA-86-8668-01	<65	Female	Stage I-II	T1-2	M0	N0
TCGA-J2-A4AD-01	<65	Female	Stage I-II	T1-2	NA	N0
TCGA-55-8299-01	<65	Female	Stage I-II	T1-2	NA	N0
TCGA-55-7913-01	<65	Female	Stage I-II	T1-2	NA	N0
TCGA-MP-A4TJ-01	<65	Female	Stage I-II	T1-2	M0	N0
TCGA-44-6145-01	<65	Female	Stage I-II	T1-2	M0	N0
TCGA-50-5946-02	<65	Male	Stage I-II	T1-2	NA	N0
TCGA-50-5946-01	<65	Male	Stage I-II	T1-2	NA	N0
TCGA-MN-A4N5-01	<65	Male	Stage I-II	T1-2	M0	N0
TCGA-50-8457-01	<65	Female	Stage I-II	T1-2	M0	N0
TCGA-L9-A443-01	<65	Female	Stage I-II	T1-2	NA	N0
TCGA-95-7043-01	<65	Female	Stage I-II	T1-2	NA	N0

TCGA-55-8085-01	<65	Male	Stage I-II	T1-2	M0	N0
TCGA-86-8669-01	<65	Male	Stage I-II	T1-2	M0	N0
TCGA-55-7903-01	<65	Male	Stage I-II	T1-2	NA	N0
TCGA-44-2655-01	>=65	Female	Stage I-II	T1-2	M0	N0
TCGA-73-4662-01	>=65	Female	Stage I-II	T1-2	M0	N0
TCGA-97-A4M2-01	>=65	Male	Stage I-II	T1-2	M0	N0
TCGA-J2-A4AG-01	>=65	Female	Stage I-II	T1-2	NA	N0
TCGA-L4-A4E6-01	>=65	Male	Stage I-II	T1-2	M0	N0
TCGA-50-5942-01	>=65	Female	Stage I-II	T1-2	M0	N0
TCGA-95-7947-01	>=65	Male	Stage I-II	T1-2	M0	N0
TCGA-44-A4SU-01	>=65	Female	Stage I-II	T1-2	NA	N0
TCGA-NJ-A55R-01	>=65	Male	Stage I-II	T1-2	NA	N0
TCGA-S2-AA1A-01	>=65	Female	Stage I-II	T1-2	M0	N0
TCGA-49-AARR-01	>=65	Male	Stage I-II	T1-2	NA	N0
TCGA-55-7725-01	>=65	Female	Stage I-II	T1-2	NA	N0
TCGA-44-2661-01	>=65	Female	Stage I-II	T1-2	M0	N0
TCGA-50-5944-01	>=65	Female	Stage I-II	T1-2	M0	N0
TCGA-97-A4M3-01	>=65	Female	Stage I-II	T1-2	M0	N0
TCGA-55-8203-01	>=65	Female	Stage I-II	T1-2	M0	N0
TCGA-69-7763-01	>=65	Male	Stage I-II	T1-2	M0	N0
TCGA-NJ-A4YQ-01	>=65	Female	Stage I-II	T1-2	M0	N0
TCGA-35-4122-01	>=65	Male	Stage I-II	T1-2	M0	N0
TCGA-86-7953-01	>=65	Female	Stage I-II	T1-2	M0	N0
TCGA-67-3770-01	>=65	Female	Stage I-II	T1-2	M0	N0
TCGA-05-4389-01	>=65	Male	Stage I-II	T1-2	M0	N0
TCGA-99-AA5R-01	>=65	Female	Stage I-II	T1-2	M0	N0
TCGA-MP-A4TH-01	>=65	Female	Stage I-II	T1-2	M0	N0
TCGA-55-7281-01	>=65	Female	Stage I-II	T1-2	M0	N0
TCGA-91-6828-01	>=65	Male	Stage I-II	T1-2	M0	N0
TCGA-69-7980-01	>=65	Female	Stage I-II	T1-2	M0	N0
TCGA-93-A4JO-01	>=65	Male	Stage I-II	T1-2	NA	N0
TCGA-55-7911-01	>=65	Female	Stage I-II	T1-2	NA	N0
TCGA-55-A492-01	>=65	Female	Stage I-II	T1-2	NA	N0
TCGA-44-7659-01	>=65	Male	Stage I-II	T1-2	NA	N0
TCGA-44-3919-01	>=65	Female	Stage I-II	T1-2	M0	N0
TCGA-95-7944-01	>=65	Male	Stage I-II	T1-2	M0	N0
TCGA-69-8255-01	>=65	Male	Stage I-II	T1-2	M0	N0
TCGA-55-A4DG-01	>=65	Male	Stage I-II	T1-2	NA	N0
TCGA-38-7271-01	>=65	Female	Stage I-II	T1-2	M0	N0
TCGA-49-4486-01	>=65	Male	Stage I-II	T1-2	M0	N0
TCGA-97-8179-01	>=65	Male	Stage I-II	T1-2	M0	N0
TCGA-49-4487-01	>=65	Female	Stage I-II	T1-2	M0	N0
TCGA-95-8039-01	>=65	Male	Stage I-II	T1-2	NA	N0
TCGA-97-7941-01	>=65	Female	Stage I-II	T1-2	NA	N0
TCGA-55-7573-01	>=65	Female	Stage I-II	T1-2	NA	N0
TCGA-55-7726-01	>=65	Female	Stage I-II	T1-2	NA	N0
TCGA-44-A47G-01	>=65	Female	Stage I-II	T1-2	M0	N0
TCGA-44-A4SS-01	>=65	Male	Stage I-II	T1-2	M0	N0
TCGA-55-8208-01	>=65	Female	Stage I-II	T1-2	M0	N0
TCGA-55-7995-01	>=65	Female	Stage I-II	T1-2	M0	N0
TCGA-50-8460-01	>=65	Male	Stage I-II	T1-2	M0	N0
TCGA-97-A4M7-01	>=65	Male	Stage I-II	T1-2	M0	N0
TCGA-49-4488-01	>=65	Female	Stage I-II	T1-2	NA	N0

TCGA-O1-A52J-01	>=65	Female	Stage I-II	T1-2	NA	N0
TCGA-78-7162-01	>=65	Male	Stage I-II	T1-2	M0	N0
TCGA-69-7764-01	>=65	Male	Stage I-II	T1-2	M0	N0
TCGA-MP-A4TA-01	>=65	Female	Stage I-II	T1-2	M0	N0
TCGA-91-8497-01	>=65	Female	Stage I-II	T1-2	NA	N0
TCGA-55-8621-01	>=65	Female	Stage I-II	T1-2	NA	N0
TCGA-93-7348-01	>=65	Female	Stage I-II	T1-2	NA	N0
TCGA-97-7546-01	>=65	Female	Stage I-II	T1-2	NA	N0
TCGA-97-7938-01	>=65	Female	Stage I-II	T1-2	NA	N0
TCGA-93-7347-01	>=65	Female	Stage I-II	T1-2	NA	N0
TCGA-91-8499-01	>=65	Female	Stage I-II	T1-2	NA	N0
TCGA-55-6987-01	>=65	Male	Stage I-II	T1-2	M0	N0
TCGA-78-8655-01	>=65	Female	Stage I-II	T1-2	M0	N0
TCGA-44-3398-01	>=65	Female	Stage I-II	T1-2	M0	N0
TCGA-67-3771-01	>=65	Female	Stage I-II	T1-2	M0	N0
TCGA-J2-A4AE-01	>=65	Female	Stage I-II	T1-2	NA	N0
TCGA-86-A456-01	>=65	Female	Stage I-II	T1-2	M0	N0
TCGA-49-4514-01	>=65	Female	Stage I-II	T1-2	M0	N0
TCGA-55-8090-01	>=65	Male	Stage I-II	T1-2	M0	N0
TCGA-55-A57B-01	>=65	Female	Stage I-II	T1-2	M0	N0
TCGA-91-6835-01	>=65	Female	Stage I-II	T1-2	M0	N0
TCGA-55-A491-01	>=65	Female	Stage I-II	T1-2	NA	N0
TCGA-97-A4M5-01	>=65	Male	Stage I-II	T1-2	M0	N0
TCGA-50-6673-01	>=65	Female	Stage I-II	T1-2	M0	N0
TCGA-50-5935-01	>=65	Female	Stage I-II	T1-2	M0	N0
TCGA-55-A4DF-01	>=65	Male	Stage I-II	T1-2	NA	N0
TCGA-78-7159-01	<65	Female	Stage I-II	T1-2	M0	NA
TCGA-44-5645-01	<65	Female	Stage I-II	T1-2	M0	NA
TCGA-44-6147-01	>=65	Female	Stage I-II	T1-2	M0	NA
TCGA-44-3917-01	<65	Female	Stage I-II	T1-2	M0	N0
TCGA-75-5146-01	<65	Male	Stage I-II	T1-2	M0	N0
TCGA-75-6206-01	<65	Male	Stage I-II	T1-2	M0	N0
TCGA-75-7025-01	<65	Male	Stage I-II	T1-2	M0	N0
TCGA-05-4420-01	<65	Male	Stage I-II	T1-2	M0	N0
TCGA-75-5147-01	<65	Female	Stage I-II	T1-2	M0	N0
TCGA-75-7027-01	<65	Male	Stage I-II	T1-2	M0	N0
TCGA-49-AARQ-01	<65	Female	Stage I-II	T1-2	NA	N0
TCGA-95-7948-01	<65	Female	Stage I-II	T1-2	M0	N0
TCGA-69-7973-01	<65	Female	Stage I-II	T1-2	M0	N0
TCGA-44-2666-01	<65	Male	Stage I-II	T1-2	M0	N0
TCGA-86-8358-01	<65	Male	Stage I-II	T1-2	M0	N0
TCGA-78-7535-01	<65	Male	Stage I-II	T1-2	M0	N0
TCGA-71-6725-01	<65	Female	Stage I-II	T1-2	M0	N0
TCGA-05-4417-01	<65	Female	Stage I-II	T1-2	M0	N0
TCGA-44-2668-01	<65	Male	Stage I-II	T1-2	M0	N0
TCGA-44-5644-01	<65	Female	Stage I-II	T1-2	M0	N0
TCGA-NJ-A4YP-01	<65	Male	Stage I-II	T1-2	M0	N0
TCGA-67-6215-01	<65	Female	Stage I-II	T1-2	M0	N0
TCGA-91-6836-01	<65	Female	Stage I-II	T1-2	NA	N0
TCGA-80-5611-01	<65	Male	Stage I-II	T1-2	M0	N0
TCGA-78-8662-01	<65	Female	Stage I-II	T1-2	M0	N0
TCGA-62-A46R-01	<65	Female	Stage I-II	T1-2	M0	N0
TCGA-44-8117-01	<65	Female	Stage I-II	T1-2	M0	N0

TCGA-55-A493-01	<65	Female	Stage I-II	T1-2	M0	N0
TCGA-55-7576-01	<65	Male	Stage I-II	T1-2	M0	N0
TCGA-55-8302-01	<65	Male	Stage I-II	T1-2	NA	N0
TCGA-64-5781-01	<65	Female	Stage I-II	T1-2	M0	N0
TCGA-97-8175-01	<65	Female	Stage I-II	T1-2	M0	N0
TCGA-55-8510-01	<65	Female	Stage I-II	T1-2	NA	N0
TCGA-78-7542-01	<65	Male	Stage I-II	T1-2	M0	N0
TCGA-35-3615-01	<65	Male	Stage I-II	T1-2	M0	N0
TCGA-38-4626-01	<65	Female	Stage I-II	T1-2	M0	N0
TCGA-86-8585-01	<65	Male	Stage I-II	T1-2	M0	N0
TCGA-55-8616-01	<65	Female	Stage I-II	T1-2	M0	N0
TCGA-05-4390-01	<65	Female	Stage I-II	T1-2	M0	N0
TCGA-44-8120-01	<65	Male	Stage I-II	T1-2	M0	N0
TCGA-86-8073-01	<65	Male	Stage I-II	T1-2	M0	N0
TCGA-55-6985-01	<65	Female	Stage I-II	T1-2	NA	N0
TCGA-55-8301-01	<65	Male	Stage I-II	T1-2	NA	N0
TCGA-05-4430-01	<65	Female	Stage I-II	T1-2	M0	N0
TCGA-97-8177-01	<65	Female	Stage I-II	T1-2	M0	N0
TCGA-44-2656-01	<65	Male	Stage I-II	T1-2	M0	N0
TCGA-55-6971-01	<65	Female	Stage I-II	T1-2	NA	N0
TCGA-55-8087-01	<65	Female	Stage I-II	T1-2	NA	N0
TCGA-64-5778-01	<65	Male	Stage I-II	T1-2	M0	N0
TCGA-64-5774-01	<65	Male	Stage I-II	T1-2	M0	N0
TCGA-78-7163-01	<65	Male	Stage I-II	T1-2	M0	N0
TCGA-97-A4M0-01	<65	Female	Stage I-II	T1-2	M0	N0
TCGA-44-4112-01	<65	Female	Stage I-II	T1-2	M0	N0
TCGA-71-8520-01	<65	Female	Stage I-II	T1-2	M0	N0
TCGA-86-8673-01	<65	Male	Stage I-II	T1-2	M0	N0
TCGA-55-A494-01	<65	Female	Stage I-II	T1-2	NA	N0
TCGA-44-7662-01	<65	Male	Stage I-II	T1-2	NA	N0
TCGA-86-7955-01	<65	Male	Stage I-II	T1-2	M0	N0
TCGA-78-7143-01	<65	Female	Stage I-II	T1-2	M0	N0
TCGA-91-6847-01	<65	Female	Stage I-II	T1-2	NA	N0
TCGA-86-A4P7-01	<65	Female	Stage I-II	T1-2	M0	N0
TCGA-55-6642-01	<65	Male	Stage I-II	T1-2	NA	N0
TCGA-44-7671-01	<65	Male	Stage I-II	T1-2	M0	N0
TCGA-55-7574-01	<65	Female	Stage I-II	T1-2	M0	N0
TCGA-49-AAR2-01	<65	Male	Stage I-II	T1-2	NA	N0
TCGA-55-7728-01	<65	Female	Stage I-II	T1-2	NA	N0
TCGA-L9-A8F4-01	<65	Female	Stage I-II	T1-2	NA	N0
TCGA-62-A46P-01	>=65	Male	Stage I-II	T1-2	M0	N0
TCGA-78-7152-01	>=65	Male	Stage I-II	T1-2	M0	N0
TCGA-NJ-A4YG-01	>=65	Male	Stage I-II	T1-2	M0	N0
TCGA-78-7153-01	>=65	Female	Stage I-II	T1-2	M0	N0
TCGA-55-1592-01	>=65	Male	Stage I-II	T1-2	M0	N0
TCGA-44-2662-01	>=65	Male	Stage I-II	T1-2	M0	N0
TCGA-62-A46O-01	>=65	Female	Stage I-II	T1-2	M0	N0
TCGA-97-7937-01	>=65	Male	Stage I-II	T1-2	NA	N0
TCGA-78-7540-01	>=65	Female	Stage I-II	T1-2	M0	N0
TCGA-86-8075-01	>=65	Female	Stage I-II	T1-2	M0	N0
TCGA-38-4625-01	>=65	Female	Stage I-II	T1-2	M0	N0
TCGA-91-6831-01	>=65	Male	Stage I-II	T1-2	NA	N0
TCGA-49-4501-01	>=65	Female	Stage I-II	T1-2	M0	N0

TCGA-05-4249-01	>=65	Male	Stage I-II	T1-2	M0	N0
TCGA-78-7633-01	>=65	Male	Stage I-II	T1-2	M0	N0
TCGA-MP-A4SV-01	>=65	Male	Stage I-II	T1-2	M0	N0
TCGA-97-7547-01	>=65	Female	Stage I-II	T1-2	NA	N0
TCGA-55-8096-01	>=65	Female	Stage I-II	T1-2	NA	N0
TCGA-05-4422-01	>=65	Male	Stage I-II	T1-2	M0	N0
TCGA-86-7954-01	>=65	Female	Stage I-II	T1-2	M0	N0
TCGA-05-4382-01	>=65	Male	Stage I-II	T1-2	M0	N0
TCGA-78-7155-01	>=65	Male	Stage I-II	T1-2	M0	N0
TCGA-05-5715-01	>=65	Female	Stage I-II	T1-2	M0	N0
TCGA-44-7661-01	>=65	Female	Stage I-II	T1-2	M0	N0
TCGA-50-5049-01	>=65	Male	Stage I-II	T1-2	M0	N0
TCGA-97-7552-01	>=65	Male	Stage I-II	T1-2	NA	N0
TCGA-55-8514-01	>=65	Female	Stage I-II	T1-2	NA	N0
TCGA-05-4426-01	>=65	Male	Stage I-II	T1-2	M0	N0
TCGA-50-6592-01	>=65	Female	Stage I-II	T1-2	M0	N0
TCGA-69-7979-01	>=65	Female	Stage I-II	T1-2	NA	N0
TCGA-50-5066-01	>=65	Male	Stage I-II	T1-2	M0	N0
TCGA-78-7537-01	>=65	Male	Stage I-II	T1-2	M0	N0
TCGA-55-6972-01	>=65	Male	Stage I-II	T1-2	M0	N0
TCGA-38-4631-01	>=65	Female	Stage I-II	T1-2	M0	N0
TCGA-50-6590-01	>=65	Female	Stage I-II	T1-2	M0	N0
TCGA-50-5066-02	>=65	Male	Stage I-II	T1-2	M0	N0
TCGA-44-7660-01	>=65	Male	Stage I-II	T1-2	NA	N0
TCGA-44-6775-01	>=65	Female	Stage I-II	T1-2	NA	N0
TCGA-67-3774-01	>=65	Female	Stage I-II	T1-2	M0	N0
TCGA-62-A46S-01	>=65	Male	Stage I-II	T1-2	M0	N0
TCGA-55-8511-01	>=65	Female	Stage I-II	T1-2	NA	N0
TCGA-55-8207-01	>=65	Male	Stage I-II	T1-2	NA	N0
TCGA-44-A479-01	>=65	Female	Stage I-II	T1-2	NA	N0
TCGA-05-4405-01	>=65	Female	Stage I-II	T1-2	M0	N0
TCGA-55-6986-01	>=65	Female	Stage I-II	T1-2	M0	N0
TCGA-55-8091-01	>=65	Male	Stage I-II	T1-2	NA	N0
TCGA-97-8172-01	>=65	Female	Stage I-II	T1-2	M0	N0
TCGA-50-5931-01	>=65	Female	Stage I-II	T1-2	M0	N0
TCGA-38-4630-01	>=65	Female	Stage I-II	T1-2	M0	N0
TCGA-NJ-A55A-01	>=65	Female	Stage I-II	T1-2	M0	N0
TCGA-MP-A5C7-01	>=65	Female	Stage I-II	T1-2	M0	N0
TCGA-05-4403-01	>=65	Male	Stage I-II	T1-2	M0	N0
TCGA-55-7815-01	>=65	Male	Stage I-II	T1-2	NA	N0
TCGA-55-8614-01	>=65	Male	Stage I-II	T1-2	NA	N0
TCGA-55-7724-01	>=65	Female	Stage I-II	T1-2	NA	N0
TCGA-93-8067-01	>=65	Male	Stage I-II	T1-2	NA	N0
TCGA-62-A46V-01	>=65	Female	Stage I-II	T1-2	M0	N0
TCGA-44-A47A-01	>=65	Female	Stage I-II	T1-2	NA	N0
TCGA-91-6829-01	>=65	Male	Stage I-II	T1-2	NA	N0
TCGA-50-6597-01	>=65	Female	Stage I-II	T1-2	M0	N0
TCGA-44-A47B-01	>=65	Male	Stage I-II	T1-2	M0	N0
TCGA-73-4658-01	>=65	Female	Stage I-II	T1-2	M0	N0
TCGA-38-A44F-01	>=65	Male	Stage I-II	T1-2	M0	N0
TCGA-97-A4LX-01	>=65	Male	Stage I-II	T1-2	M0	N0
TCGA-73-7499-01	>=65	Female	Stage I-II	T1-2	M0	N0
TCGA-67-3772-01	>=65	Female	Stage I-II	T1-2	M0	N0

TCGA-05-4433-01	>=65	Male	Stage I-II	T1-2	M0	N0
TCGA-67-3773-01	>=65	Female	Stage I-II	T1-2	M0	N0
TCGA-62-A470-01	>=65	Male	Stage I-II	T1-2	M0	N0
TCGA-69-7761-01	>=65	Male	Stage I-II	T1-2	NA	N0
TCGA-50-5939-01	>=65	Male	Stage I-II	T1-2	M0	N0
TCGA-55-8204-01	>=65	Female	Stage I-II	T1-2	NA	N0
TCGA-91-8496-01	<65	Female	Stage I-II	T1-2	NA	NA
TCGA-44-2657-01	>=65	Female	Stage I-II	T1-2	M0	NA
TCGA-44-6777-01	>=65	Female	Stage I-II	T1-2	NA	NA
TCGA-69-7765-01	<65	Male	Stage I-II	T3-4	NA	N0
TCGA-44-7670-01	<65	Female	Stage I-II	T1-2	M0	N1-3
TCGA-NJ-A55O-01	<65	Female	Stage I-II	T1-2	M0	N1-3
TCGA-05-5428-01	<65	Male	Stage I-II	T1-2	M0	N1-3
TCGA-55-5899-01	<65	Male	Stage I-II	T1-2	M0	N1-3
TCGA-78-8640-01	<65	Male	Stage I-II	T1-2	M0	N1-3
TCGA-69-8253-01	<65	Female	Stage I-II	T1-2	NA	N1-3
TCGA-44-7669-01	<65	Male	Stage I-II	T1-2	NA	N1-3
TCGA-86-8074-01	<65	Female	Stage I-II	T1-2	M0	N1-3
TCGA-55-A48X-01	<65	Female	Stage I-II	T1-2	M0	N1-3
TCGA-49-AAQV-01	<65	Female	Stage I-II	T1-2	NA	N1-3
TCGA-38-4627-01	<65	Female	Stage I-II	T1-2	M0	N1-3
TCGA-44-2659-01	>=65	Female	Stage I-II	T1-2	M0	N1-3
TCGA-91-6830-01	>=65	Female	Stage I-II	T1-2	NA	N1-3
TCGA-55-7914-01	>=65	Female	Stage I-II	T1-2	NA	N1-3
TCGA-86-6851-01	>=65	Female	Stage I-II	T1-2	M0	N1-3
TCGA-L9-A50W-01	>=65	Male	Stage I-II	T1-2	NA	N1-3
TCGA-53-7626-01	>=65	Female	Stage I-II	T1-2	M0	N1-3
TCGA-91-A4BD-01	>=65	Male	Stage I-II	T1-2	NA	N1-3
TCGA-50-5055-01	>=65	Female	Stage I-II	T1-2	M0	N1-3
TCGA-86-A4D0-01	<65	Male	Stage I-II	T1-2	M0	N0
TCGA-55-7910-01	<65	Female	Stage I-II	T1-2	M0	N0
TCGA-86-8280-01	<65	Female	Stage I-II	T1-2	M0	N0
TCGA-MP-A4TE-01	<65	Male	Stage I-II	T1-2	NA	N0
TCGA-MP-A4TF-01	<65	Female	Stage I-II	T1-2	M0	N0
TCGA-91-A4BC-01	<65	Male	Stage I-II	T1-2	NA	N0
TCGA-97-8174-01	>=65	Male	Stage I-II	T1-2	M0	N0
TCGA-55-A48Y-01	>=65	Male	Stage I-II	T1-2	M0	N0
TCGA-86-7713-01	>=65	Male	Stage I-II	T1-2	M0	N0
TCGA-73-4677-01	>=65	Male	Stage I-II	T1-2	M0	N0
TCGA-78-7539-01	>=65	Female	Stage I-II	T1-2	M0	N0
TCGA-55-8205-01	>=65	Female	Stage I-II	T1-2	M0	N0
TCGA-55-A490-01	>=65	Male	Stage I-II	T1-2	NA	N0
TCGA-55-6978-01	>=65	Male	Stage I-II	T1-2	NA	N0
TCGA-75-6212-01	<65	Female	Stage I-II	T1-2	M0	N1-3
TCGA-75-5125-01	<65	Male	Stage I-II	T1-2	M0	N1-3
TCGA-73-4676-01	<65	Male	Stage I-II	T1-2	M0	N1-3
TCGA-86-8279-01	<65	Male	Stage I-II	T1-2	M0	N1-3
TCGA-86-8674-01	<65	Male	Stage I-II	T1-2	M0	N1-3
TCGA-44-6779-01	<65	Female	Stage I-II	T1-2	NA	N1-3
TCGA-49-4510-01	<65	Female	Stage I-II	T1-2	M0	N1-3
TCGA-86-6562-01	<65	Male	Stage I-II	T1-2	M0	N1-3
TCGA-MP-A4SW-01	<65	Male	Stage I-II	T1-2	M0	N1-3
TCGA-44-2665-01	<65	Female	Stage I-II	T1-2	M0	N1-3

TCGA-55-1596-01	<65	Male	Stage I-II	T1-2	M0	N1-3
TCGA-L9-A743-01	<65	Male	Stage I-II	T1-2	M0	N1-3
TCGA-MP-A4TK-01	<65	Female	Stage I-II	T1-2	NA	N1-3
TCGA-55-6979-01	<65	Female	Stage I-II	T1-2	M0	N1-3
TCGA-78-7150-01	<65	Male	Stage I-II	T1-2	M0	N1-3
TCGA-50-5068-01	<65	Female	Stage I-II	T1-2	NA	N1-3
TCGA-69-7978-01	<65	Male	Stage I-II	T1-2	NA	N1-3
TCGA-MN-A4N1-01	<65	Male	Stage I-II	T1-2	M0	N1-3
TCGA-55-8508-01	<65	Female	Stage I-II	T1-2	NA	N1-3
TCGA-49-4505-01	<65	Female	Stage I-II	T1-2	M0	N1-3
TCGA-MP-A4SY-01	<65	Male	Stage I-II	T1-2	M0	N1-3
TCGA-95-7567-01	<65	Male	Stage I-II	T1-2	M0	N1-3
TCGA-55-6975-01	<65	Male	Stage I-II	T1-2	M0	N1-3
TCGA-86-8054-01	<65	Male	Stage I-II	T1-2	M0	N1-3
TCGA-95-A4VN-01	<65	Female	Stage I-II	T1-2	M0	N1-3
TCGA-86-8278-01	<65	Female	Stage I-II	T1-2	M0	N1-3
TCGA-53-A4EZ-01	<65	Male	Stage I-II	T1-2	NA	N1-3
TCGA-62-A471-01	<65	Male	Stage I-II	T1-2	M0	N1-3
TCGA-49-6744-01	<65	Female	Stage I-II	T1-2	NA	N1-3
TCGA-05-5423-01	>=65	Male	Stage I-II	T1-2	M0	N1-3
TCGA-05-4397-01	>=65	Male	Stage I-II	T1-2	M0	N1-3
TCGA-38-4628-01	>=65	Female	Stage I-II	T1-2	M0	N1-3
TCGA-05-4427-01	>=65	Female	Stage I-II	T1-2	M0	N1-3
TCGA-J2-8192-01	>=65	Female	Stage I-II	T1-2	NA	N1-3
TCGA-73-4668-01	>=65	Female	Stage I-II	T1-2	M0	N1-3
TCGA-05-4432-01	>=65	Male	Stage I-II	T1-2	M0	N1-3
TCGA-78-7147-01	>=65	Female	Stage I-II	T1-2	M0	N1-3
TCGA-95-8494-01	>=65	Male	Stage I-II	T1-2	M0	N1-3
TCGA-49-4506-01	>=65	Female	Stage I-II	T1-2	M0	N1-3
TCGA-MP-A4T4-01	>=65	Female	Stage I-II	T1-2	M0	N1-3
TCGA-05-5425-01	>=65	Male	Stage I-II	T1-2	M0	N1-3
TCGA-78-8660-01	>=65	Male	Stage I-II	T1-2	M0	N1-3
TCGA-L9-A7SV-01	>=65	Male	Stage I-II	T1-2	M0	N1-3
TCGA-49-AAR3-01	>=65	Male	Stage I-II	T1-2	NA	N1-3
TCGA-49-6742-01	>=65	Male	Stage I-II	T1-2	M0	N1-3
TCGA-86-7711-01	>=65	Male	Stage I-II	T1-2	M0	N1-3
TCGA-78-7148-01	>=65	Male	Stage I-II	T1-2	M0	N1-3
TCGA-55-6984-01	>=65	Female	Stage I-II	T1-2	M0	N1-3
TCGA-95-7562-01	>=65	Male	Stage I-II	T1-2	M0	N1-3
TCGA-55-6712-01	>=65	Male	Stage I-II	T1-2	NA	N1-3
TCGA-MP-A4TI-01	>=65	Male	Stage I-II	T1-2	M0	N1-3
TCGA-86-8671-01	>=65	Female	Stage I-II	T1-2	M0	N1-3
TCGA-67-6217-01	>=65	Female	Stage I-II	T1-2	M0	N1-3
TCGA-64-5815-01	>=65	Male	Stage I-II	T1-2	M0	N1-3
TCGA-50-5932-01	>=65	Male	Stage I-II	T1-2	M0	N1-3
TCGA-55-7907-01	>=65	Male	Stage I-II	T1-2	NA	N1-3
TCGA-55-6982-01	>=65	Female	Stage I-II	T1-2	M0	N1-3
TCGA-86-8055-01	>=65	Male	Stage I-II	T1-2	M0	N1-3
TCGA-55-6983-01	>=65	Male	Stage I-II	T1-2	M0	N1-3
TCGA-78-7166-01	>=65	Male	Stage I-II	T1-2	M0	N1-3
TCGA-69-8254-01	>=65	Male	Stage I-II	T1-2	NA	N1-3
TCGA-73-A9RS-01	<65	Male	Stage I-II	T3-4	M0	N0
TCGA-49-6767-01	<65	Female	Stage I-II	T3-4	NA	N0

TCGA-44-7667-01	<65	Female	Stage I-II	T3-4	NA	N0
TCGA-95-7039-01	<65	Female	Stage I-II	T3-4	NA	N0
TCGA-86-A4JF-01	<65	Male	Stage I-II	T3-4	M0	N0
TCGA-78-8648-01	<65	Female	Stage I-II	T3-4	M0	N0
TCGA-86-8672-01	<65	Male	Stage I-II	T3-4	M0	N0
TCGA-69-A59K-01	<65	Female	Stage I-II	T3-4	M0	N0
TCGA-49-AAR9-01	<65	Male	Stage I-II	T3-4	NA	N0
TCGA-55-8506-01	<65	Female	Stage I-II	T3-4	NA	N0
TCGA-91-7771-01	<65	Male	Stage I-II	T3-4	NA	N0
TCGA-44-6146-01	<65	Male	Stage I-II	T3-4	M0	N0
TCGA-38-4629-01	>=65	Male	Stage I-II	T3-4	M0	N0
TCGA-50-8459-01	>=65	Male	Stage I-II	T3-4	M0	N0
TCGA-78-7161-01	>=65	Female	Stage I-II	T3-4	M0	N0
TCGA-67-4679-01	>=65	Male	Stage I-II	T3-4	M0	N0
TCGA-J2-8194-01	>=65	Female	Stage I-II	T3-4	NA	N0
TCGA-62-A472-01	>=65	Male	Stage I-II	T3-4	M0	N0
TCGA-62-8397-01	>=65	Female	Stage I-II	T3-4	M0	N0
TCGA-05-4424-01	>=65	Male	Stage I-II	T3-4	M0	N0
TCGA-55-8619-01	>=65	Female	Stage I-II	T3-4	NA	N0
TCGA-69-7760-01	>=65	Male	Stage I-II	T3-4	M0	N0
TCGA-44-8119-01	>=65	Male	Stage I-II	T3-4	M0	N0
TCGA-55-7284-01	>=65	Male	Stage I-II	T3-4	NA	N0
TCGA-55-8092-01	>=65	Male	Stage I-II	T3-4	NA	N0
TCGA-55-8513-01	>=65	Female	Stage I-II	T3-4	NA	N0
TCGA-69-8453-01	>=65	Male	Stage I-II	T3-4	NA	N0
TCGA-62-8395-01	>=65	Female	Stage I-II	T3-4	M0	N0
TCGA-55-7994-01	>=65	Male	Stage I-II	T3-4	NA	N0
TCGA-50-6593-01	<65	Female	Stage III-IV	T1-2	M0	N1-3
TCGA-55-6981-01	<65	Female	Stage III-IV	T1-2	M0	N1-3
TCGA-44-6774-01	<65	Female	Stage III-IV	T1-2	M0	N1-3
TCGA-64-1679-01	<65	Female	Stage III-IV	T1-2	M0	N1-3
TCGA-86-A4P8-01	<65	Female	Stage III-IV	T1-2	NA	N1-3
TCGA-86-7714-01	<65	Female	Stage III-IV	T1-2	M0	N1-3
TCGA-55-8505-01	<65	Male	Stage III-IV	T1-2	NA	N1-3
TCGA-49-6761-01	>=65	Female	Stage III-IV	T1-2	NA	N1-3
TCGA-55-7727-01	>=65	Male	Stage III-IV	T1-2	NA	N1-3
TCGA-MP-A4T6-01	>=65	Female	Stage III-IV	T1-2	NA	N1-3
TCGA-MP-A4TC-01	>=65	Male	Stage III-IV	T1-2	M0	N1-3
TCGA-49-6743-01	>=65	Female	Stage III-IV	T1-2	NA	N1-3
TCGA-55-A48Z-01	<65	Female	Stage III-IV	T1-2	NA	N1-3
TCGA-64-1678-01	>=65	Female	Stage III-IV	T1-2	M0	N0
TCGA-50-5045-01	<65	Female	Stage III-IV	T1-2	M0	N1-3
TCGA-75-6214-01	<65	Female	Stage III-IV	T1-2	M0	N1-3
TCGA-50-5051-01	<65	Female	Stage III-IV	T1-2	M0	N1-3
TCGA-50-5930-01	<65	Male	Stage III-IV	T1-2	M0	N1-3
TCGA-49-AAR4-01	<65	Male	Stage III-IV	T1-2	NA	N1-3
TCGA-78-7220-01	<65	Female	Stage III-IV	T1-2	M0	N1-3
TCGA-44-5643-01	<65	Male	Stage III-IV	T1-2	M0	N1-3
TCGA-69-7974-01	<65	Female	Stage III-IV	T1-2	NA	N1-3
TCGA-MP-A4T9-01	<65	Female	Stage III-IV	T1-2	NA	N1-3
TCGA-50-5941-01	<65	Female	Stage III-IV	T1-2	M0	N1-3
TCGA-62-8398-01	<65	Male	Stage III-IV	T1-2	M0	N1-3
TCGA-50-5936-01	<65	Male	Stage III-IV	T1-2	M0	N1-3

TCGA-91-6848-01	<65	Male	Stage III-IV	T1-2	NA	N1-3
TCGA-35-5375-01	<65	Male	Stage III-IV	T1-2	M0	N1-3
TCGA-64-5779-01	<65	Male	Stage III-IV	T1-2	M0	N1-3
TCGA-62-8399-01	<65	Male	Stage III-IV	T1-2	M0	N1-3
TCGA-05-4384-01	>=65	Male	Stage III-IV	T1-2	M0	N1-3
TCGA-73-4659-01	>=65	Male	Stage III-IV	T1-2	M0	N1-3
TCGA-95-A4VP-01	>=65	Female	Stage III-IV	T1-2	M0	N1-3
TCGA-05-5420-01	>=65	Male	Stage III-IV	T1-2	M0	N1-3
TCGA-55-6970-01	>=65	Female	Stage III-IV	T1-2	NA	N1-3
TCGA-MP-A4T8-01	>=65	Male	Stage III-IV	T1-2	M0	N1-3
TCGA-55-1594-01	>=65	Male	Stage III-IV	T1-2	M0	N1-3
TCGA-78-7536-01	>=65	Male	Stage III-IV	T1-2	M0	N1-3
TCGA-49-4512-01	>=65	Female	Stage III-IV	T1-2	NA	N1-3
TCGA-62-A46Y-01	>=65	Female	Stage III-IV	T1-2	M0	N1-3
TCGA-38-6178-01	>=65	Female	Stage III-IV	T1-2	M1	N1-3
TCGA-MP-A4TD-01	>=65	Male	Stage III-IV	T1-2	M0	N1-3
TCGA-78-7146-01	>=65	Female	Stage III-IV	T1-2	M0	N1-3
TCGA-62-8402-01	>=65	Female	Stage III-IV	T1-2	M0	N1-3
TCGA-44-3396-01	>=65	Female	Stage III-IV	T1-2	M0	N1-3
TCGA-95-A4VK-01	>=65	Female	Stage III-IV	T1-2	M0	N1-3
TCGA-50-6595-01	>=65	Female	Stage III-IV	T1-2	M0	N1-3
TCGA-50-5072-01	>=65	Male	Stage III-IV	T1-2	M0	N1-3
TCGA-91-6849-01	>=65	Female	Stage III-IV	T1-2	NA	N1-3
TCGA-64-1677-01	>=65	Female	Stage III-IV	T1-2	M0	N1-3
TCGA-97-8547-01	>=65	Female	Stage III-IV	T1-2	NA	N1-3
TCGA-49-6745-01	>=65	Male	Stage III-IV	T1-2	M0	N1-3
TCGA-97-7554-01	>=65	Female	Stage III-IV	T1-2	M0	N1-3
TCGA-NJ-A4YI-01	>=65	Female	Stage III-IV	T1-2	M0	N1-3
TCGA-73-4675-01	<65	Male	Stage III-IV	T3-4	M0	N1-3
TCGA-97-8176-01	<65	Male	Stage III-IV	T3-4	M0	N1-3
TCGA-49-4507-01	>=65	Female	Stage III-IV	T3-4	M0	N1-3
TCGA-55-7227-01	>=65	Male	Stage III-IV	T3-4	NA	N1-3
TCGA-05-4250-01	>=65	Female	Stage III-IV	T3-4	M0	N1-3
TCGA-49-4490-01	<65	Female	Stage III-IV	T3-4	M0	N1-3
TCGA-86-8359-01	<65	Male	Stage III-IV	T3-4	M0	N1-3
TCGA-05-5429-01	<65	Male	Stage III-IV	T3-4	M0	N1-3
TCGA-55-8615-01	>=65	Male	Stage III-IV	T3-4	NA	N1-3
TCGA-05-4418-01	>=65	Male	Stage III-IV	T3-4	M0	N1-3
TCGA-99-8025-01	>=65	Female	Stage III-IV	T3-4	M0	N1-3
TCGA-78-7154-01	>=65	Male	Stage III-IV	T3-4	M0	N1-3
TCGA-55-7283-01	>=65	Female	Stage III-IV	T3-4	NA	N1-3
TCGA-49-4494-01	>=65	Male	Stage III-IV	T3-4	M0	N1-3
TCGA-50-6594-01	>=65	Female	Stage III-IV	T3-4	M0	N1-3
TCGA-53-7813-01	<65	Female	Stage III-IV	T3-4	M0	N0
TCGA-86-8056-01	<65	Female	Stage III-IV	T3-4	M0	N0
TCGA-78-7149-01	>=65	Male	Stage III-IV	T3-4	M0	N0
TCGA-64-5775-01	>=65	Male	Stage III-IV	T3-4	M0	N0
TCGA-99-7458-01	>=65	Female	Stage III-IV	T3-4	M0	N0
TCGA-NJ-A7XG-01	<65	Male	Stage III-IV	T3-4	M0	N1-3
TCGA-50-5044-01	>=65	Female	Stage III-IV	T3-4	M0	N1-3
TCGA-05-4396-01	>=65	Male	Stage III-IV	T3-4	M0	N1-3
TCGA-05-4415-01	<65	Male	Stage III-IV	T3-4	M0	N1-3
TCGA-78-7158-01	<65	Female	Stage III-IV	T3-4	M0	N1-3

TCGA-62-8394-01	>=65	Female	Stage III-IV	T3-4	M0	N1-3
TCGA-50-5933-01	>=65	Male	Stage III-IV	T3-4	M0	N1-3
TCGA-05-4398-01	<65	Female	Stage III-IV	T3-4	M0	N1-3
TCGA-55-7816-01	<65	Female	Stage III-IV	NA	NA	NA
TCGA-93-A4JP-01	<65	Male	Stage III-IV	NA	M1	NA
TCGA-99-8033-01	>=65	Female	Stage III-IV	NA	M1	NA
TCGA-73-4666-01	<65	Female	Stage III-IV	T1-2	M1	N0
TCGA-55-6968-01	<65	Male	Stage III-IV	T1-2	M1	N0
TCGA-55-8512-01	<65	Male	Stage III-IV	T1-2	M1	N1-3
TCGA-55-8620-01	<65	Male	Stage III-IV	T1-2	M1	N1-3
TCGA-53-7624-01	<65	Female	Stage III-IV	T1-2	M1	N0
TCGA-55-8094-01	<65	Male	Stage III-IV	T1-2	M1	N0
TCGA-50-6591-01	<65	Female	Stage III-IV	T1-2	M1	N0
TCGA-86-7701-01	>=65	Male	Stage III-IV	T1-2	M1	N0
TCGA-73-4670-01	>=65	Female	Stage III-IV	T1-2	M1	N0
TCGA-05-4425-01	>=65	Female	Stage III-IV	T1-2	M1	N0
TCGA-93-A4JN-01	>=65	Male	Stage III-IV	T1-2	M1	N0
TCGA-MP-A4T7-01	>=65	Female	Stage III-IV	T1-2	M1	N0
TCGA-78-7167-01	>=65	Male	Stage III-IV	T1-2	M1	N0
TCGA-38-4632-01	<65	Male	Stage III-IV	T1-2	M1	N1-3
TCGA-64-1680-01	<65	Male	Stage III-IV	T1-2	M1	N1-3
TCGA-97-8171-01	>=65	Male	Stage III-IV	T1-2	M1	N1-3
TCGA-05-4402-01	<65	Female	Stage III-IV	T1-2	M1	NA
TCGA-L9-A5IP-01	<65	Female	Stage III-IV	T3-4	M1	N1-3
TCGA-78-7145-01	<65	Female	Stage III-IV	T3-4	M1	N1-3
TCGA-78-7156-01	<65	Male	Stage III-IV	T3-4	M1	N1-3
TCGA-05-4434-01	>=65	Female	Stage III-IV	T3-4	M1	N1-3
TCGA-78-7160-01	<65	Male	Stage III-IV	T3-4	M1	N1-3
